# Inhibition of PRC2 enables self-renewal of blastoid-competent naive pluripotent stem cells from chimpanzee

**DOI:** 10.1016/j.stem.2025.02.002

**Published:** 2025-02-26

**Authors:** Tao Huang (黄瑫), Arthur Radley, Ayaka Yanagida, Zhili Ren (任志丽), Francesca Carlisle, Somayyeh Tahajjodi, Dongwan Kim, Paul O’Neill, James Clarke, Madeline A. Lancaster, Zoe Heckhausen, Jingran Zhuo (卓靖然), João Pedro Agostinho de Sousa, Petra Hajkova, Ferdinand von Meyenn, Hiroo Imai, Hiromitsu Nakauchi, Ge Guo (郭歌), Austin Smith, Hideki Masaki

**Affiliations:** 1 Living Systems Institute, University of Exeter, Exeter EX4 4QD, UK; 2 Department of Veterinary Anatomy, The University of Tokyo, Tokyo 113-8657, Japan; 3 Division of Stem Cell Therapy, Institute of Medical Science, University of Tokyo, Tokyo 108-8639, Japan; 4 Stem Cell Therapy Division, Institute of Integrated Research, Institute of Science, Tokyo 113-8510, Japan; 5 University of Exeter Sequencing Facility, University of Exeter, Exeter EX4 4QD, UK; 6 Wellcome-MRC Stem Cell Institute, University of Cambridge, Cambridge CB2 0AW, UK; 7 MRC Laboratory of Molecular Biology, Cambridge Biomedical Campus, Cambridge CB2 0QH, UK; 8 MRC Laboratory of Medical Sciences (LMS), Du Cane Rd, London W12 0HS, UK; 9 Institute of Clinical Sciences, Faculty of Medicine, Imperial College London, W12 0NN, UK; 10 Department of Health Sciences and Technology, ETH Zurich, 8603 Schwerzenbach, Switzerland; 11 Department of Cellular and Molecular Biology, Center for the Evolutionary Origins of Human Behavior, Kyoto University, Inuyama, Aichi 484-8506, Japan; 12 Institute for Stem Cell Biology and Regenerative Medicine, Department of Genetics, Stanford University School of Medicine, Stanford, CA 94305, USA

## Abstract

Naive pluripotent stem cells (PSCs) are counterparts of early epiblast in the mammalian embryo. Mouse and human naive PSCs differ in self-renewal requirements and extraembryonic lineage potency. Here, we investigated the generation of chimpanzee naive PSCs. Colonies generated by resetting or reprogramming failed to propagate. We discovered that self-renewal is enabled by inhibition of Polycomb repressive complex 2 (PRC2). Expanded cells show global transcriptome proximity to human naive PSCs and embryo pre-implantation epiblast, with shared expression of a subset of pluripotency transcription factors. Chimpanzee naive PSCs can transition to multilineage competence or can differentiate into trophectoderm and hypoblast, forming tri-lineage blastoids. They thus provide a higher primate comparative model for studying pluripotency and early embryogenesis. Genetic deletions confirm that PRC2 mediates growth arrest. Further, inhibition of PRC2 overcomes a roadblock to feeder-free propagation of human naive PSCs. Therefore, excess deposition of chromatin modification H3K27me3 is an unexpected barrier to naive PSC self-renewal.

## Graphical abstract

**Graphical abstract:**
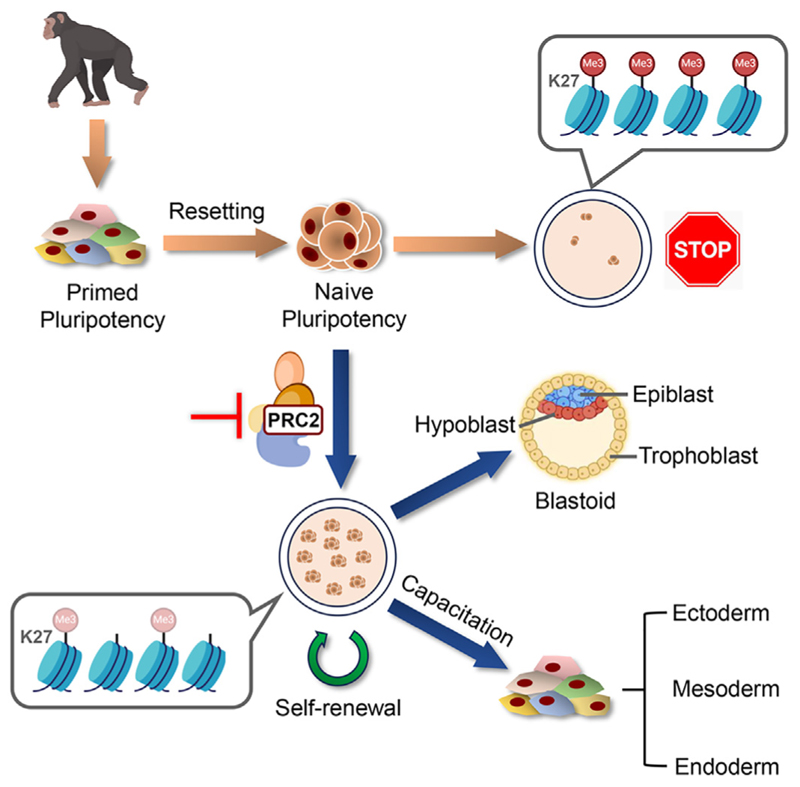

## Introduction

Embryos of eutherian mammals develop from a small group of unspecialized cells, the naive epiblast that forms a few days after fertilization within the inner cell mass (ICM) of the blastocyst. As the fount of germ line and soma, naive epiblast might be expected to show high conservation of cellular features and to govern the gene regulatory network. However, species-specific features have been noted,^1–4^ and the degree of evolutionary divergence or developmental drift^5^ has yet to be determined.

Naive pluripotent stem cell (naive PSC) lines that correspond closely to naive epiblast in the embryo have been established from mice,^6,7^ rats,^8,9^ and human.^10–12^ The signaling environment for self-renewal is similar but not identical between mouse and rat,^13^ while human naive PSCs show distinct dependencies.^14–17^ Importantly, divergence between mouse and human naive PSCs reflects distinctions in gene expression and developmental plasticity observed in the embryo. Notably, human naive PSCs and epiblast exhibit potency to form trophectoderm whereas mouse embryonic stem cells (ESCs) and epiblast are lineage restricted.^18,19^ Consequently, propagation of human but not mouse naive PSCs requires inhibition of signals that induce trophectoderm.^15^ The capacity of human naive PSCs to differentiate into trophectoderm means that they are uniquely competent to form blastocyst-like structures termed blastoids, which contain all three primary lineages and have exciting potential to model aspects of pre- and peri-implantation embryogenesis.^20–22^

Naive PSCs are thus a unique resource to dissect the early stages of primate embryogenesis and molecular regulation thereof.^22,23^ Recently, PSCs that show some naive features have been reported for marmoset^24^ and macaque,^25^ but their relatedness to either rodent or human naive PSCs is unclear. Chimpanzee (*Pan troglodytes*) is the closest extant relative of human, less than 7 million years in evolutionary distance. However, previously described chimpanzee PSCs^26,27^ do not display naive character. They are derived and maintained via stimulation of fibroblast growth factor (FGF) and transforming growth factor β (TGF-β)/nodal signaling pathways, conditions that support a stage of pluripotency termed primed.^28^ Primed PSCs are related to post-implantation embryonic disc epiblast,^4,28–31^ and they lack competence to form blastoids.

Primed PSCs have undergone a formative transition that changes developmental competence through enhancer reorganization and rewiring of the gene regulatory network.^23,32^ Consequently naive and primed PSCs are epigenomically distinct. Notably the histone modification H3K27me3, deposited by the Polycomb repressive complex 2 (PRC2) and associated with gene silencing,^33^ is very broadly distributed in naive PSCs but with few distinct peaks; whereas in primed PSCs, H3K27me3 is lower overall but higher over many gene regulatory regions.^34,35^ Gene deletions in mouse ESCs^36,37^ and human naive PSCs^35,38^ have established that PRC2 is not required for their self-renewal. In contrast, primed PSCs are dependent on PRC2 to silence somatic lineage gene expression.^38^

Here, we investigate establishment of naive PSCs from chimpanzee and explore whether self-renewal requirements, signature transcription factors (TFs), and unrestricted lineage potential of the naive state are shared hominid features or unique to humans.

## Results

### Generation and propagation of chimpanzee naive iPSCs

Ethical and practical considerations prohibit research directly on chimpanzee embryos. We therefore employed molecular reprogramming to convert somatic cells into induced PSCs (iPSCs).^39,40^ We used Sendai virus vectors or RNA delivery to reprogram blood cells or fibroblasts (see STAR Methods). As previously reported,^26,27^ chimpanzee iPSCs propagate in the same conditions as human primed PSCs and are similar in morphology and marker expression (Figure S1A).

**Figure 1:**
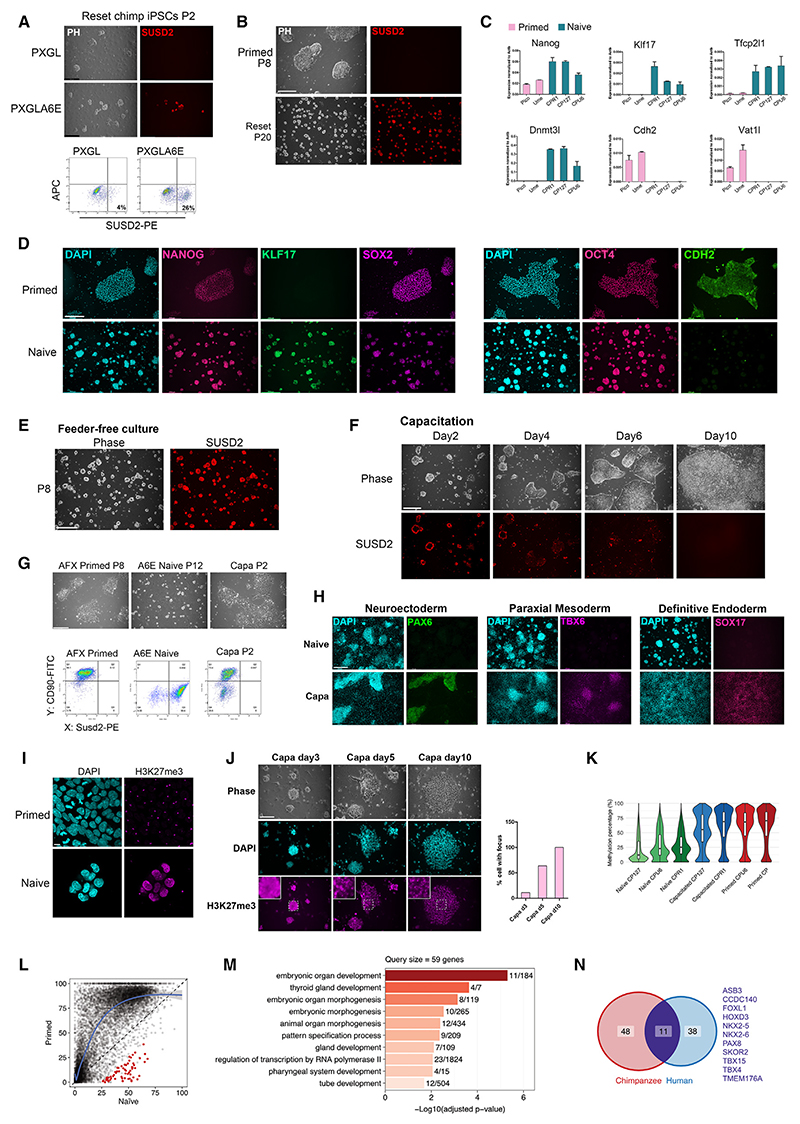
Generation and self-renewal of chimpanzee naive PSCs (A) Morphology and SUSD2 live staining of resetting chimpanzee PSCs in PXGL and PXGL-A6E after exposure to VPA. Plot shows SUSD2-PE flow cytometry at P2. (B) Live-cell staining of primed and stabilized reset PSCs for SUSD2. (C) qRT-PCR analysis of pluripotent state markers in reset and primed PSC lines. SD from three biological replicates. (D) Immunostaining of primed PSCs and reset PSCs at P18. (E) SUSD2 staining of feeder-free reset PSCs at P8. (F) Phase image and SUSD2 live staining during capacitation. (G) Phase contrast images and flow cytometry analyses for SUSD2 and CD90 of primed, reset, and capacitated PSCs. (H) Somatic lineage differentiation of capacitated chimpanzee PSCs. (I) Immunostaining of H3K27me3 in primed and reset PSCs. Scale bar: 20 μm. (J) Immunostaining of H3K27me3 during capacitation scored for nuclei with an intense focus. (K) Genome-wide methylation distribution for CpGs with ≥5× coverage. (L) Promotor methylation comparison between naive and primed cells. Red indicates promoters with significantly higher methylation in naive cells. (M) Top 10 GO biological process terms for genes associated with promoters hypermethylated in naive cells. (N) Comparison between chimpanzee and human of genes with increased promoter methylation in naive PSCs. Human genes from Guo et al.^41^ Scale bars: 278.5 μm unless otherwise indicated. See also Figure S1.

Human PSCs can be converted to naive status by transient inhibition of histone deacetylase with valproic acid and transfer to medium containing PXGL (see STAR Methods).^41^ When this protocol was applied to chimpanzee iPSCs (Table S2), we observed emergence of compact refractile colonies that expressed naive markers such as KLF17 and SUSD2 (Figure S1B). However, colonies failed to propagate beyond 2–3 passages. Rarely, naïve type cells expanded but only after a severe bottleneck that was not apparent during resetting of human PSCs. We concluded that PXGL is deficient for propagation of chimpanzee naive PSCs. We therefore added three candidate regulators: the growth factors activin and interleukin-6 (IL-6) plus the small-molecule EPZ-6438. EPZ-6438 is a competitive inhibitor of EZH2, the enyzmatic component of PRC2.^42^ In the combined medium, termed PXGL-A6E, we saw that domed colonies expressing SUSD2 propagate continuously. Cultures are initially quite heterogeneous, as in human PSC resetting^41^ (Figure 1A). Naive-type cells are readily enriched by flow cytometric sorting for SUSD2^43^ or non-adherent culture, with collection of cell clusters after 2–3 days (see STAR Methods). A single round of enrichment by either method is typically sufficient to establish relatively homogeneous populations (Figure 1B) that can be stably expanded as adherent cultures for >10 passages. Cells are passaged every 3 days with a 1:3 split ratio. Cultures are morphologically similar to human naive PSCs, although colonies are more resistant to dissociation. Reset PSCs express mRNA and protein markers indicative of naive status and lack markers of formative or primed pluripotency (Figures 1C and 1D). G-banding shows a diploid chromosome complement, with no large structural rearrangements detected in 5/6 lines analyzed (Figure S1C; Table S2). We examined *TP53*, the gene most often mutated in human PSCs.^44^ No coding changes between reset and primed PSCs were detected in RNA sequencing (RNA-seq) data from 4 lines at different passages (see STAR Methods).

In the absence of feeders, reset PSCs plated on geltrex in PXGL-A6E sustain proliferation, domed morphology, and SUSD2 expression over >10 passages with no overt differentiation (Figure 1E). Feeder-free cultures remain diploid (Figure S1D). They express naive state markers and do not exhibit post-implantation epiblast or trophectoderm markers (Figures S1E and S1F).

### Capacitation and somatic lineage differentiation

Naive PSCs require capacitation to respond to somatic lineage induction.^23,32,45–47^ Accordingly, we did not observe appreciable expression of lineage markers when inductive protocols were applied directly to reset chimpanzee cells. We transferred cells to capacitation conditions, N2B27 supplemented with XAV939,^46^ and saw emergence of flattened epithelial morphology from 3 to 4 days (Figure 1F). At 10 days cultures resemble conventional chimpanzee iPSCs and can be expanded in medium containing activin, FGF2, and XAV939 (AFX)^46^ (Figure 1G). Capacitated cells respond to lineage-specific differentiation protocols by expressing markers of definitive endoderm, paraxial mesoderm, or neuroectoderm, respectively (Figures 1H and S1G). We also observed formation in immuno-deficient mice of teratomas with representation of three germ layers (Figure S1H).

### Transcriptome identity

**Figure 2:**
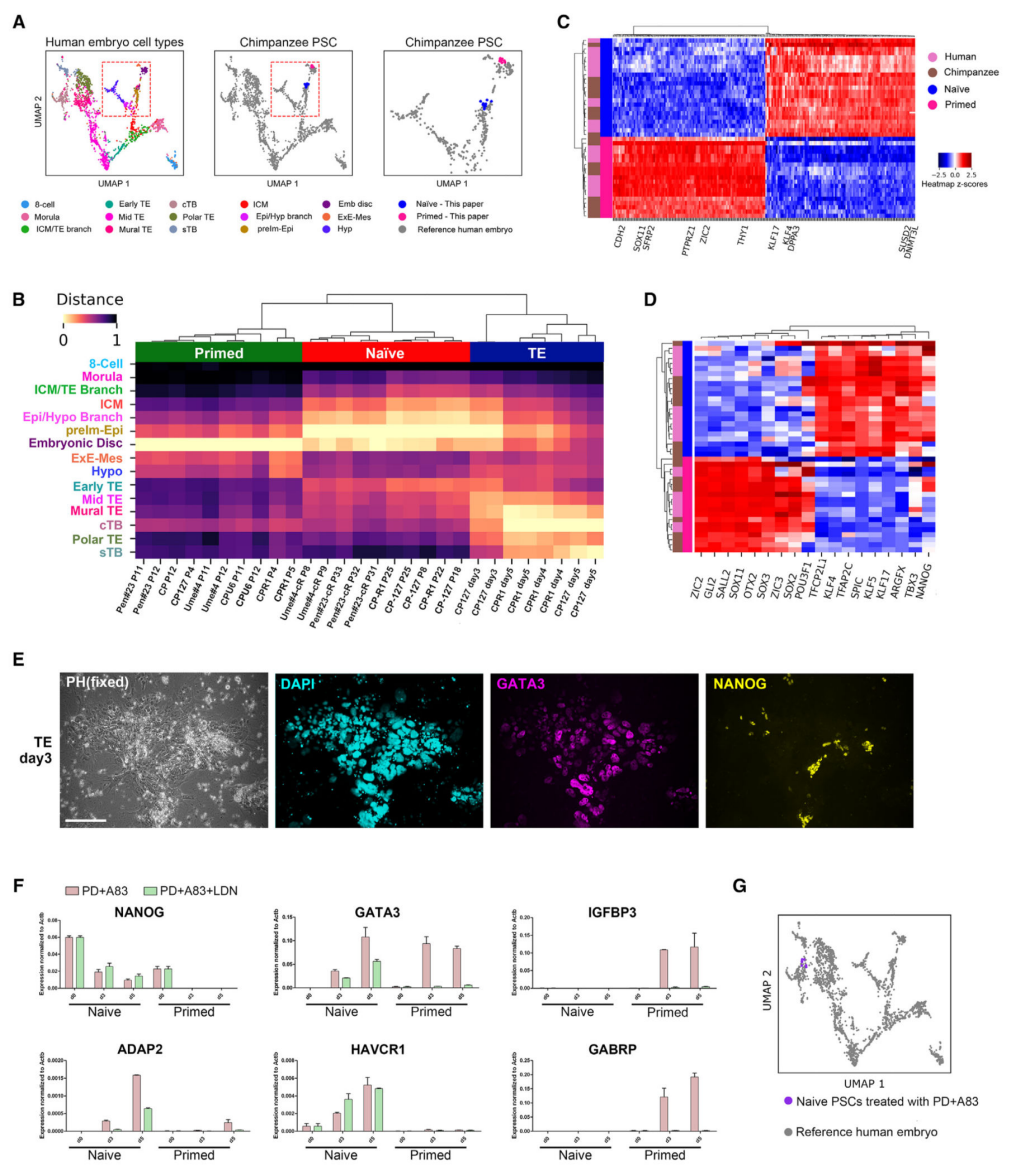
Transcriptome identity and trophectoderm differentiation of chimpanzee naive PSCs (A) Mapping of bulk RNA-seq samples for chimpanzee reset and primed PSCs onto UMAP embedding of human embryo scRNA-seq datasets.^48^ Left, embryo UMAP; center, projection of PSC samples; right, expanded ICM, hypoblast, and epiblast region. (B) Heatmap of correlation distance metrics between chimpanzee cell samples and human embryo cell types. Trophectoderm samples are from differentiation time points as in (G) below. (C) Heatmap of top 200 differentially expressed genes between naive and primed PSC samples in both chimpanzee and human. (D) Cluster map of pluripotency factor expression in human and chimpanzee naive or primed PSCs. (E) Immunostaining after treatment of naive PSCs with PD+A83 for 5 days. Scale bar: 278.5 μm. (F) qRT-PCR analysis at days 0, 3, and 5 of naive or primed PSCs differentiating in PD+A83 with or without BMP signal inhibition by LDN. SD from two biological replicates. (G) Projection onto the human embryo UMAP embedding of bulk RNA-seq samples from differentiation of two chimpanzee naive PSC lines in PD+A83 (four samples of each cell line). See also Figure S1 and Table S1.

We carried out bulk RNA-seq on primed and reset chimpanzee iPSCs. Principal-component analysis (PCA) computed using all protein-coding genes separated reset from primed samples (Figure S1I). To assess embryonic identities, we used human embryo datasets as a reference. We took advantage of a high-resolution uniform manifold approximation and projection (UMAP) embedding generated from six single-cell RNA-seq (scRNA-seq) datasets spanning from day 3 to approximately day 14.^48^ The embedding shows coherent developmental progression for 15 cell types and stages, including the pluripotent lineage from morula to ICM, pre- and post-implantation epiblast (Figure 2A). We used UMAP transformation to map PSC samples onto the embedding space. This algorithm positions query samples relative to cells in the embedding with the most similar gene expression. Human primed PSCs are placed proximal to post-implantation embryonic disc epiblast, and human naive PSCs map to the pre-implantation epiblast at E6–E7 as expected^4,18^ (Figure S1J). Chimpanzee samples exhibited similar placements; conventional PSCs overlying embryonic disc and reset PSCs located with pre-implantation naive epiblast (Figure 2A). Correlation distance metrics analysis supports these identities (Figure 2B).

We grouped human and chimpanzee samples together and applied DEseq2 to identify the top 200 differentially expressed genes between naive and primed PSCs (Table S1). The heatmap (Figure 2C) shows unambiguous separation between naive and primed states for both species. In contrast, chimpanzee and human samples are intermingled in both cluster maps, evidencing high similarity between species. We examined a curated set of pluripotency-associated TFs, including those known to be differentially expressed between pre- and post-implantation epiblast in the human embryo. These factors discriminate naive and primed PSCs for chimpanzee as for human (Figure 2D). This analysis highlights conserved candidates for the core gene regulatory networks of naive (TBX3, TFCP2L1, ARGFX, KLF17, KLF4, KLF5, SPIC, TFAP2C, NANOG) or primed (ZIC2, ZIC3, GLI2, SALL2, POU3F1, OTX2, SOX3, SOX11) PSC states.

### Epigenome features of naive pluripotency

In mouse and human, pre-implantation epiblast and naive PSCs exhibit distinctive epigenome features: activation of both X chromosomes in female cells^10,49–51^ and global DNA hypomethylation. To monitor the epigenomic status of X chromosomes, we undertook immunostaining for H3K27me3 and H2Aub, which densely coat the inactive X^52,53^. Female primed PSCs display prominent single foci of nuclear staining. To examine naive PSCs, we released EZH2 inhibition and cultured cells in PXGL-A6 for 1 passage. Staining showed homogeneous nuclear H3K27me3 at higher levels than in primed cells, as previously noted in mouse and human naive PSCs, but without foci (Figure 1I). Foci appeared during capacitation (Figure 1J). H2Aub foci were also absent in female naive PSCs (Figure S1K).

For quantitative assessment of DNA methylation, we used liquid chromatography-tandem mass spectrometry (LC-MS/MS).^54^ The measurements show total 5 mC is greatly reduced in chimpanzee naive PSCs compared with primed PSCs, in line with assays on human PSCs, and is largely restored upon capacitation (Figure S1L). Whole-genome bisulfite sequencing confirms the genome-wide changes in methylation levels with a substantial reduction at most promoter regions in the naive state (Figure 1K). As in human naive PSCs, loss of methylation extends to imprinted regions (Figure S1M). In contrast to this general trend, a small group of promoters gain methylation in the naive state (Figure 1L). These loci show Gene Ontology (GO) enrichment for developmental processes (Figure 1M). They partially overlap with hypermethylated genes previously identified in human naive PSCs^14,41^ (Figure 1N), suggesting that gain of methylation is non-random.

### Differentiation to trophoblast and hypoblast

Human naive PSCs have been shown to differentiate into trophectoderm.^18,55^ This was unexpected as trophectoderm forms before specification of epiblast in the embryo and is not produced by mouse ESCs. We investigated whether chimpanzee naive PSCs have plasticity to make trophectoderm. We applied ERK and nodal pathway inhibition (PD+A83) that induce trophectoderm from human naive PSCs.^18^ At 3 days, we saw GATA3 immunostaining and detected trophectoderm markers by qRT-PCR (Figures 2E and 2F). By contrast, conventional chimpanzee PSCs do not show appreciable expression of trophectoderm-specific markers but upregulate amnion markers. Bone morpho-genetic protein (BMP) signal inhibition with LDN193189, which blocks amnion differentiation, has no effect on naive PSC differentiation to trophoblast (Figure 2F). Feeder-free chimpanzee naive PSCs similarly generate trophectoderm (Figure S2A). Bulk RNA-seq analysis of cultures differentiated in PD+A83 for 3 or 5 days shows relatedness to trophoblast by projection on the human embryo UMAP (Figure 2G) and correlation distance metrics (Figure 2B).

We transferred trophectoderm cells generated by 3 days of PD+A83 treatment into medium for human cytotrophoblast stem cell expansion^55^ and saw robust expansion of epithelial colonies that could be continuously propagated (Figure S2B). We induced further differentiation along the trophoblast lineage and observed morphology and markers of syncytiotrophoblast and extra-villous trophoblast (Figures S2C and S2D).

We also tested hypoblast differentiation of chimpanzee naive PSCs, using conditions recently established for human.^22^ Treatment with PD03 for 48 h, followed by exchange to FGF, A83, and XAV939 for 48 h, yielded clusters of cells positive for hypoblast marker FOXA2 (Figure S2E).

### Formation of tri-lineage blastoids

**Figure 3:**
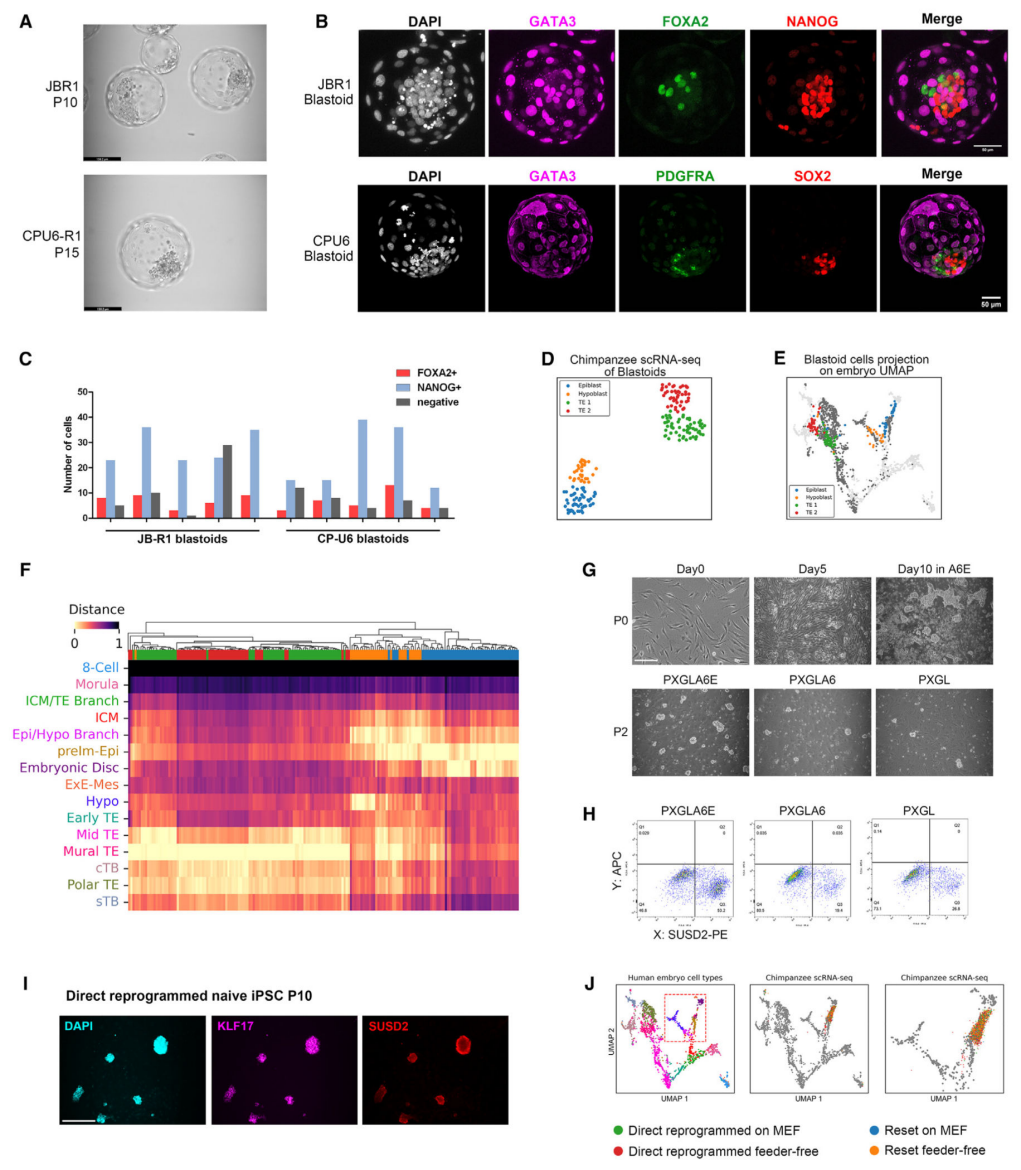
Chimpanzee naive PSCs form blastoids and can be generated by direct reprogramming (A) Phase images of day 4 blastoids from indicated lines. Scale bar: 139 μm. (B) Confocal images of blastoids immunostained for indicated lineage markers. Scale bar: 50 μm. (C) Numbers of hypoblast (FOXA2)- or epiblast (NANOG)-positive inner cells in individual day 4 blastoids. Double-negative cells may be trophoblast or nascent hypoblast. (D) UMAP with Leiden clustering of day 4 blastoid scRNA-seq data. (E) Projection of blastoid scRNA-seq samples on human embryo UMAP. (F) Comparison of blastoids and human embryo datasets by correlation distance metrics. Sample colors as in (D). (G) Morphology of naive iPSC colonies emerging during direct reprogramming and expansion after passage in indicated media. (H) SUSD2 flow cytometry of directly reprogrammed cells after 2 passages in indicated medium. (I) Immunostaining of KLF17 and SUSD2 in directly reprogrammed naive iPSCs at P10. Scale bar: 144 μm. (J) Projection onto the human embryo UMAP embedding of scRNA-seq data from chimpanzee naive PSCs cultured on MEF or feeder free. See also Figure S2.

We investigated whether chimpanzee naive PSCs can form blastocyst-like structures, blastoids.^20,21^ We adapted a protocol for human blastoid formation, clustering cells in PALY (PD, A83, LPA, and Y-27632), then transferring to A83 with oleoyl-L-alpha-lysophosphatidic acid [LPA], and finally N2B27. Cavitated structures with internal cells formed, albeit with different efficiencies between lines (Figures 3A, 3B, and S2F). Immunostaining of day 4 blastoids from 3 different lines shows trophectoderm marker GATA3 throughout the outer layer, with epiblast and hypoblast markers in the inner population (Figures 3B and S2F). Up to 80% of cysts expressing GATA3 and OCT4 also express hypoblast marker GATA4 in some inner cells (Figure S2G). Mature hypoblast marker FOXA2 is present in 10% to 20% of inner cells (Figure 3C). Hypoblast is also under-represented in human blastoids.^22^

We used SMART-seq2^56^ scRNA-seq to validate the composition of day 4 blastoids relative to human embryo datasets. We manually excised most of the mural trophectoderm to avoid over-representation. Analysis of 185 cells from 14 blastoids, using the Scanpy workflow, yielded 4 clusters, annotated as epiblast-, hypoblast-, and trophectoderm-related (Figures 3D and S2H). Trophectoderm clusters show negligible expression of amnion markers GABRP or IGFBP3.^57^ Projection onto the human embryo UMAP shows relatedness to blastocyst-stage cell types, substantiated by correlation distance metrics (Figures 3E and 3F). Chimpanzee blastoids do not contain significant numbers of cells that are unannotated or have inappropriate lineage features, unlike some reports of human and monkey blastoids.^57^ The hypoblast-like population includes cells that overly the epiblast-hypoblast branchpoint cluster in the human embryo, aligning with the suggestion that naive PSCs revert to a pre-lineage ICM state prior to hypoblast and trophectoderm differentiation.^22^

### Reprogramming chimpanzee fibroblasts directly to naive PSC status

Human somatic cells can be reprogrammed into naive PSCs using RNAs.^14^ In initial trials with chimpanzee fibroblasts, we saw that, as with resetting, naive-type colonies emerge but cannot be propagated. We repeated the experiment, transferring transfected cells on day 7 to either AFX medium for conventional iPSC generation or to PXGL, PXGL-A6, or PXGL-A6E. Flattened epithelial colonies appeared in AFX from 10 days onward, whereas refractile dome-shaped colonies arose in all three PXGL conditions. Only in PXGL-A6E do naive-type colonies expand after passaging (Figure 3G). Most colonies are SUSD2 positive, and serial passaging is sufficient to establish a stable iPSC culture (Figures 3H and 3I). Expression of naive pluripotency TFs (Figure S2I) and differentiation into trophectoderm in PD+A83 (Figures S2J and S2K) corroborate naive iPSC status.

We performed 10× Genomics single-cell transcriptome analysis on reprogrammed and reset naive PSCs cultured with and without feeders. In UMAP visualization, using all protein-coding genes, reprogrammed cells form a single cluster, with feeder and feeder-free PSCs intermingled (Figure S2L). Key naive markers are expressed throughout the populations. Hierarchical clustering using markers of pluripotency stage from the bulk RNA-seq analyses (Figure S2M) shows extensive intermixing, indicating similar transcriptome states regardless of method of generation or maintenance. The analysis confirms the presence of naive markers and negligible expression of primed or trophectoderm markers. All four samples of chimpanzee naive PSCs overlie pre-implantation epiblast when projected onto the human embryo UMAP (Figures 3J and S2N).

### Accumulation of H3K27me3 impedes chimpanzee naive PSC self-renewal

**Figure 4:**
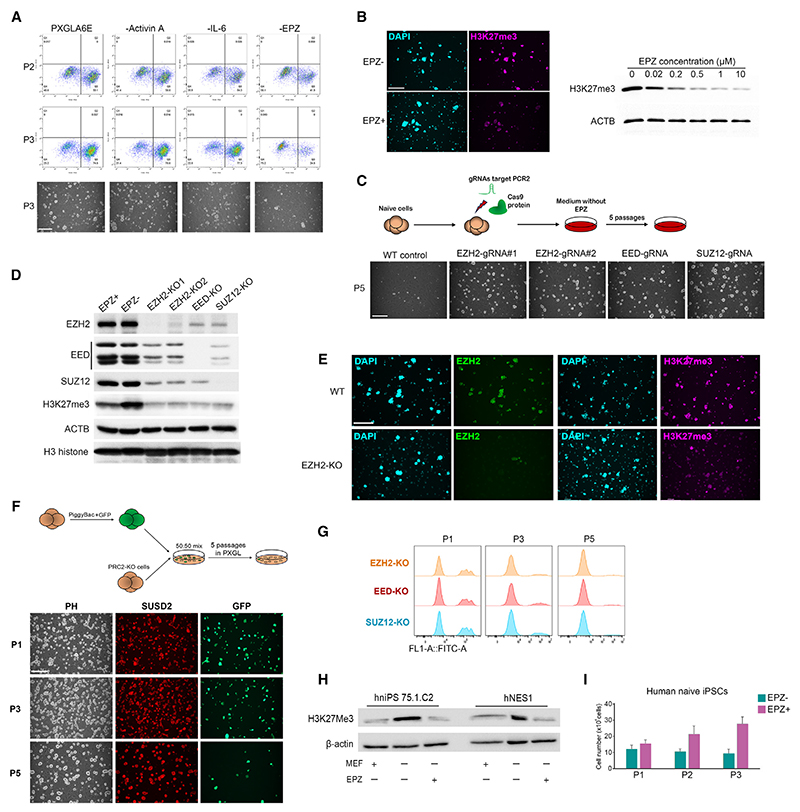
Reduced activity of PRC2 sustains self-renewal of chimpanzee and human naive PSCs (A) SUSD2 flow cytometry and images after transfer to indicated conditions for 2 or 3 passages (P). Scale bar: 278.5 μm. (B) Immunostaining for H3K27me3 and immunoblotting after culture in indicated concentrations of EPZ for 7 days. Scale bar: 278.5 μm. (C) Schematic of PRC2 knockouts and images of knockout colonies. (D) Immunoblotting analysis of knockout cells. (E) Immunostaining of H3K27me3 and EZH2 in *EZH2*-KO cells. Scale bar: 278.5 μm. (F) Schematic of cell competition experiment and images of co-cultures showing SUSD2 staining and GFP expression at P1, P3, and P5. (G) Flow cytometry detection of GFP in P1, P3, and P5 mixed cultures for each knockout. (H) Immunoblot of H3K27me3 in human naive PSC lines after three passages in indicated conditions. EPZ 0.2 μM. (I) Cell counts over sequential passages for feeder-free human naive iPSCs in PXGL with or without EPZ. SD from triplicate cultures. See also Figures S3 and S4.

We examined the roles of A6E components by withdrawing individual factors and monitoring SUSD2+ cells by flow cytometry. Removing activin or IL-6 has little effect. In contrast, withdrawal of EPZ results in dramatic diminution in the SUSD2+ population with growth arrest after 3 passages (Figure 4A). Cells in PXGL plus EPZ only continue to expand and retain naive colony morphology and SUSD2 expression (Figure S3A; Table S2). They express naive markers and remain responsive to trophectoderm induction (Figure S3B). We conclude that EPZ is the critical factor enabling propagation of chimpanzee naive PSCs.

Recently a formulation termed 4CL has been proposed to support naive PSCs from macaque.^58,59^ In addition to MEK and tankyrase inhibitors, 4CL contains broad-spectrum epigenome-modifying agents. We transferred chimpanzee naive PSCs to 4CL. The majority of cells differentiated or died within 2 passages, and we were unable to propagate a naive phenotype. We also attempted resetting directly into 4CL medium but saw few refractile domed colonies, and those could not be expanded (Figures S3C and S3D).

EPZ of 0.5 μM substantially reduces but does not eliminate H3K27me3 (Figure 4B). We confirmed that 0.5 μM EPZ is sufficient to sustain naive PSC expansion, similarly to the 1.0 μM used in the preceding experiments (Figure S3E). Alternative EZH2-selective SAM-competitive inhibitors, GSK126 and GSK343, can replace EPZ and sustain chimpanzee naive iPSC propagation both immediately after resetting and in established cultures (Figure S3F).

Another inhibitor, UNC1999, caused collapse of naive colonies after 2 passages with widespread cell death. To confirm that PRC2 is the relevant target of EPZ, we generated knockouts (KOs) using CRISPR-Cas9. We used previously validated guide RNAs (gRNAs) to disrupt *EZH2* exons 7 or 15.^38,60^ We also targeted *EED* and *SUZ12*, encoding core structural components of PRC2. After PRC2, targeting cells expanded without EPZ (Figure 4C). Immunoblotting and immunostaining confirmed the loss of PRC2 proteins and a massive depletion in H3K27me3 signal for each KO (Figures 4D, 4E, and S3G). Expanded KO cells remain SUSD2 positive and express NANOG and KLF17 (Figures 4F and S3H). They do not upregulate GATA3 in PXGL but do respond to trophectoderm induction in PD+A83 (Figure S3I). We repeated the KOs and assayed in competitive co-culture with parental naive PSCs that express GFP constitutively (Figure S3J). Targeted and GFP-expressing cells were mixed 50:50 and cultured feeder free without EPZ. The proportion of GFP-negative KO colonies increased with each passage such that they comprised almost the entire population by P5 (Figures 4F and 4G). These results confirm the EPZ-independence and growth advantage of naive PSCs lacking PRC2.

### EZH2 inhibition supports self-renewal of human naive PSCs

We investigated whether EZH2 inhibition may be beneficial for human naive PSC expansion. On MEF feeder layers, PXGL is sufficient for long-term expansion.^61^ However, in the absence of feeders, growth slows down and typically stalls after 3 passages. Cells remain undifferentiated, but the population does not increase. By immunoblotting, we detected a marked increase in H3K27me3. Culture in EPZ (0.1–0.2 μM) reduced this to a similar level as on feeders (Figure 4H). EPZ or GSK126 maintained expansion of feeder-free cultures of embryo-derived (HNES1)^12^ or directly reprogrammed^14^ human naive iPSCs for at least 10 passages (Figures 4I and S4A). Feeder-free cultures in PXGL-E express markers of naive status and do not upregulate GATA3 (Figures S4B–S4D). They retain the ability to differentiate into trophectoderm when treated with PD+A83 (Figures S4E and S4F). scRNA-seq analysis of feeder-free PXGL-E-expanded cells confirmed relatedness to pre-implantation epiblast (Figure S4G) with a naive pluripotency marker profile indistinguishable from parallel cultures in PXGL on feeders (Figure S4H).

## Discussion

These findings demonstrate that naive PSCs can reliably be established from chimpanzee. Overall, chimpanzee naive PSCs show similar properties to human naive PSCs, including global gene expression profile and TF repertoire. In common with human, chimpanzee naive PSCs exhibit unrestricted lineage potential with competence to differentiate into trophectoderm and hypoblast and an ability to form self-organizing blastoids. They thus offer a unique comparative model of hominid early embryogenesis. Strikingly, however, while the PXGL culture environment developed for human naive PSCs is effective for the initial generation of chimpanzee naive PSCs, it is not adequate to sustain self-renewal. Continuous expansion requires partial inhibition of PRC2. PRC2 inhibition is also beneficial for propagation of human naive PSCs, but the more stringent requirement in chimpanzee illustrates the potential of comparative studies to provide new insight. Indeed, a recent functional genomics screen identified 75 genes with varying effects on proliferation between chimpanzee and human.^62^

PRC2 mediates deposition of the histone modification H3K27me3, which is classically associated with gene repression.^33^ In contrast to primed PSCs and somatic cell types, H3K27me3 is pervasive at moderate levels over the entire genome in naive PSCs, although only rarely enriched at regulatory regions.^34,35,63^ An earlier report indicated that human naive PSCs withstand PRC2 ablation,^38^ unlike primed PSCs.^60^ Two recent studies proposed that PRC2 may be important in naive PSCs to suppress trophectoderm differentiation.^35,64^ However, those observations were under differentiation permissive conditions, not in PXGL. We did not detect trophectoderm in chimpanzee or human naive PSCs treated with EZH2 inhibitors or ablated for PRC2 while maintained in PXGL.

In mouse ESCs, even after complete inhibition or elimination of PRC2, H3K27me3 profile and developmental potential can be fully restored.^37,65^ Thus, the likelihood of long-term epigenetic consequences of EZH2 inhibition for primate naive PSCs may be low. Furthermore, effective sub-micromolar concentrations of the inhibitor do not erase H3K27me3 but prevent over-accumulation.

PRC2 levels and global H3K27me3 are higher in naive than in primed PSCs.^35^ The finding that excess H3K27me3 can be limiting for expansion of primate naive PSCs is therefore unexpected, introducing a new axis of regulation in PSC self-renewal. Future studies will dissect why H3K27me3 increases and how it suppresses naive PSC propagation. We speculate that impaired self-renewal may be due to either a global dampening of gene expression or to silencing of specific proliferation or cell survival factors. The greater dependency on PRC2 inhibition for propagation of chimpanzee compared with human naive PSCs points to evolutionary drift^5^ in pluripotency regulation. Studies in other non-human primates will determine whether PRC2 activity impedes naive PSC derivation more generally.^66^ A further goal will be to delineate the relevance for epiblast regulation in embryos.

### Limitations of the study

Tissue samples from higher primates are difficult to source due to ethical and regulatory considerations, and we have yet to evaluate whether PXGL-E may be effective in other great apes. Our study demonstrates chromosomal stability, but improvements in the chimpanzee genome sequence will enable sequence-based analyses. Loss of imprinting is an anomalous feature of both human and chimpanzee naive PSCs. We are reliant on human embryo data for an *in vivo* reference because transcriptome data from chimpanzee embryos are not available. Lack of H3K27me3 and H2Aub foci may indicate XaXa status, but biallelic gene expression remains to be demonstrated because of the unavailability of BAC probes for RNA-FISH studies. Embryo chimaera or uterine transfer experiments cannot be carried out in chimpanzee, and thus *in vivo* developmental potential cannot be tested.

## Resource Availability

### Lead contact

Further information and requests for resources and reagents should be directed to and will be fulfilled by the lead contact, Hideki Masaki (masakih.sct@tmd.ac.jp).

### Materials availability

All stable reagents generated in this study are available from the lead contact without restriction except for chimpanzee cells and genomic material, which are available subject to Institutional MTA and CITES regulations.

## Star★Methods

Detailed methods are provided in the online version of this paper and include the following:

- KEY RESOURCES TABLE
- EXPERIMENTAL MODEL AND STUDY PARTICIPANT DETAILS
  ○ Chimpanzee samples
  ○ Mouse strains
  ○ Cell Culture
- METHOD DETAILS
  ○ Cell culture
  ○ Differentiation
  ○ Immunostaining
  ○ Chromosome analysis
  ○ Immunoblotting
  ○ Reverse transcription PCR
  ○ Flow cytometry
  ○ Transcriptome sequencing
  ○ Whole genome bisulfite sequencing (WGBS)
- QUANTIFICATION AND STATISTICAL ANALYSIS
  ○ qRT-PCR and cell number counts
  ○ Quantification of 5-methylcytosine by LC-MS/MS
  ○ Transcriptome analyses

## Star★Methods

### Key Resources Table

**Table:** 

REAGENT or RESOURCE	SOURCE	IDENTIFIER
Antibodies		
Rabbit polyclonal anti-KLF17	Atlas Antibodies	Cat# HPA024629, RRID: AB_1668927
Goat polyclonal anti-NANOG	R&D System	Cat# AF1997, RRID:AB_355097
Rabbit polyclonal anti-NANOG	Abcam	Cat# ab21624,RRID:AB_446437
Mouse monoclonal anti-NANOG	Cell Signaling Technology	Cat# 4893,RRID: AB_10548762
Rabbit polyclonal anti-Oct-4	Cell Signaling Technology	Cat# 2750,RRID: AB_823583
Mouse monoclonal anti-OCT4	Santa Cruz	Cat# sc-5279, RRID: AB_628051
Mouse monoclonal anti-Sox2	Santa Cruz	Cat# sc-365823, RRID: AB_10842165
Goat anti-Sox2	Santa Cruz	Cat# sc17320,RRID: AB_2286684
Rat monoclonal anti-Sox2	eBiosicence	Cat #14-9811-90, RRID: AB-11219070
Mouse monoclonal anti CDH2	Biolegend	Cat# 350807,RRID: AB_10983063
Mouse monoclonal anti PAX6	Millipore	Cat# MAB5554, RRID: AB_570718
Rabbit polyclonal anti TBX6	Abcam	Cat# ab38883,RRID: AB_778274
Goat polyclonal anti-SOX17	R&D System	Cat# AF1924,RRID: AB_355060
Rabbit polyclonal anti H3K27me3	Millipore	Cat# 07-449,RRID: AB_310624
Rabbit monoclonal anti-Gata3	Abcam	Cat# ab199428,RRID: AB_2819013
Mouse monoclonal anti-Gata3	Thermo Fisher Scientific	Cat# MA1-028,RRID: AB_2536713
Rat monoclonal anti-Gata-4	Thermo Fisher Scientific	Cat# 14-9980-82, RRID: AB_763541
Rabbit polyclonal anti-HNF3beta/FOXA2	Millipore	Cat# 07-633,RRID: AB_390153
Rabbit monoclonal anti EZH2	Cell Signaling Technology	Cat# 5246,RRID: AB_10694683
Anti-EED	Cell Signaling Technologies	Cat# 85322S,RRID: AB_2923355
Anti-SUZ12	Cell Signaling Technologies	Cat# 3737S,RRID: AB_2196850
Mouse monoclonal anti ACTB	Cell Signaling Technology	Cat# 3700,RRID: AB_2242334
Rabbit monoclonal anti H3 Histon	Abcam	Cat# ab176842,RRID: AB_2493104
Anti-H2AK119ub	Cell Signaling Technology	Cat# 8240,RRID: AB_10891618
Mouse monoclonal anti TFAP2C	Santa Cruz	Cat# sc12762,RRID: AB_667770
Mouse monoclonal anti SDC1	Biolegend	Cat# 356501,RRID: AB_2561789
Rabbit monoclonal anti CGB1	Abcam	Cat# ab131170,RRID: AB_11156864
Anti-HLA-G	Abcam	Cat# ab239342
Rabbit monoclonal anti-KRT7	Abcam	Cat# ab181598,RRID: AB_2783822
Donkey anti-Rat Alexa Fluor 488	Thermo Fisher Scientific	Cat# A-21208,RRID: AB_2535794
Donkey anti-Mouse Alexa Fluor 546	Thermo Fisher Scientific	Cat# A-10036,RRID: AB_11180613
Donkey anti- Rabbit Alexa Fluor 647	Thermo Fisher Scientific	Cat# A-31573,RRID: AB_2536183
Donkey anti-Mouse Alexa Fluor 647	Molecular Probes	Cat# A-31571,RRID: AB_162542
Donkey anti-Rabbit Alexa Fluor 405	Abcam	Cat# A48258,RRID: AB_2890547
Donkey anti-Goat Alexa Fluor 555	Thermo Fisher Scientific	Cat # A-21432,RRID: AB_2535853
Donkey anti-Rabbit Alexa Fluor 488	Thermo Fisher Scientific	Cat # A-21206,RRID: AB_2535792
Donkey anti-Mouse Alexa Fluor 647	Thermo Fisher Scientific	Cat# A-31571,RRID: AB_162542
Anti-SUSD2-PE	Biolegend	Cat# 327406,RRID: AB_940654
Anti-SUSD2-APC	Biolegend	Cat# 327408,RRID: AB_2561888
Anti-CD90-FITC	BioLegend	Cat# 328107,RRID: AB_893429
Anti-FOXA2	R&D	Cat# AF2400,RRID: AB_2294104
Anti-PDGFRA	abcam	Cat# ab203491 RRID: AB_2892065
Bacterial and virus strains		
SeVdp(KOSM302L)	https://doi.org/10.1016/j.scr.2017.06.011	N/A
Chemicals, peptides, and recombinant proteins		
MEK inhibitor PD0325901	abcr; FUJIFILM Wako	Cat# AB 253775; Cat# 162-25291
Tankyrase inhibitor XAV939	Cell Guidance Systems; TCI	Cat# SMS38-200; Cat#X0077
PKC inhibitor Gö6983	Bio-Techne; FUJIFILM Wako	Cat# 2285; Cat# 074-06443
Rock inhibitor Y-27632	Merck Chemicals; FUJIFILM Wako	Cat# 688000; Cat# 034-24024
LIF	Qkine; PeproTech	Cat# Qk036; Cat# 300-05
Activin-A	Qkine; PeproTech	Cat# Qk005; Cat# AF-120-14E
EPZ-6438 (Tazemetostat)	MedChemExpress	Cat# HY-13803
IL6	ORIENTAL YEAST	Cat# 4708200
Activin receptor inhibitor A83-01	Generon; FUJIFILM Wako	Cat# A12358-50; Cat# 039-24111
BMP receptor inhibitor LDN-193189	Axon Medchem	Cat# Axon 1509
Oleoyl-L-alpha-lysophosphatidic acid (LPA)	Sigma	Cat# L7260
A83-01	Generon; FUJIFILM Wako	Cat# A12358-50; Cat# 039-24111
FGF2	Qkine; Katayama Chemical	Cat# Qk002; Cat# 160-0010-3
GSK inhibitor CHIR99021	abcr	Cat# AB 253776
VPA	Sigma	Cat# P4543
EGF	Peprotech	Cat# AF-100-15
Forskolin	Merck	Cat# 344282
Neuregulin-1	CST	Cat# 26941
GSK126	ApexBio	Cat# A3446
GSK343	Sigma	Cat# SML-0766
UNC1999	Selleck	Cat# S7165
StemRNA 3rd Gen Reprogramming Kit	StemRNA	Cat# 00-0076
Lipofectamine RNAiMAX Transfection Reagent	Thermo	Cat# 13778150
TrueCut Cas9 Protein v2	ThermoFisher Scientific	Cat# A36498
N2B27	Made in-house	N/A
NDiff 227	Takara	Cat# Y40002
Accutase	Millipore	Cat# SCR005
TrypLE™ Express Enzyme	Thermo Fisher Scientific	Cat# 12604021
D-MEM high Glucose	FUJIFILM Wako	Cat# 044-29765
Opti-MEM I Reduced Serum Medium	Gibco	Cat# 31985062
0.25% trypsin-EDTA	Thermo Fisher Scientific	Cat# 25200056
MEM-alpha	Thermo Fisher Scientific	Cat# 11900016
Fetal bovine serum	Corning	Cat# 35-010-CV
Geltrex	Thermo Fisher Scientific	Cat# A1413302
Gelatin from porcine skin	Sigma	Cat# G1890
Critical commercial assays		
StemRNA 3^rd^ Gen Reprogramming Kit	ReproCell	Cat# 00-0076
Neon 10μL transfection kit	Thermo Fisher Scientific	Cat# MPK1096
Deposited data		
scRNAseq	Yanagida et al.^20^	GEO: GSE171820
Raw sequence data	This paper	SRA: PRJNA1086168
Bulk RNA-seq	This paper	GEO: GSE264735
scRNA-seq	This paper	GEO: GSE278810
Whole genome bisulfite sequencing	This paper	GEO: GSE282157
Experimental models: Cell lines		
Chimpanzee fibroblasts and blood cells	This paper	Great Ape Information Network (GAIN) (https://shigen.nig.ac.jp/gain/top.jsp) ID: 0306, 0439, 0439, 0027
Mouse embryo fibroblasts	Prepared in-house	N/A
Leo#9 iPSC	This paper	N/A
Pen#23 iPSC	This paper	N/A
Pico#16 iPSC	This paper	N/A
Ume#6 iPSC	This paper	N/A
Ja-C12 iPSC	This paper	N/A
Leo#9-cR naïve iPSC	This paper	N/A
Pen#23-cR naïve iPSC	This paper	N/A
Pico#16-cR naïve iPSC	This paper	N/A
Ume#6-cR naïve iPSC	This paper	N/A
CPR1 naïve iPSC	This paper	N/A
CP127 naïve iPSC	This paper	N/A
CPU6 R1 naïve iPSC	This paper	N/A
TCP1 naïve iPSC	This paper	N/A
JB-R1 naïve iPSC	This paper	N/A
HNES1	Guo et al.^12^	hPSCreg: CAMe001-A
HNES1-GATA3-cas9	Guo et al.^18^	N/A
hniPSC 75.1c2	Bredenkamp et al.^14^	hPSCreg: CSCIi002-A
Experimental models: Organisms/strains		
NSG mice	Jackson Laboratory	cat#005557
Oligonucleotides		
TrueGuide tracrRNA	Thermo Fisher Scientific	Cat# A35508
cpEZH2-gRNAe7-F	TCTTCTGCTGTGCCCTTATC	N/A
cpEZH2-gRNAe7-R	GATAAGGGCACAGCAGAAGA	N/A
cpEZH2-gRNAset-F	ATTGCTGGCACCATCTGACG	N/A
cpEZH2-gRNAset-R	CGTCAGATGGTGCCAGCAAT	N/A
cpEEDKO-gRNAe5-F	ATGGCTCGTATTGCTATCAT	N/A
cpEEDKO-gRNAe5-R	ATGATAGCAATACGAGCCAT	N/A
cpSUZ12KO-gRNA-F	TATGGAAATACAGACGATTG	N/A
cpSUZ12KO-gRNA-R	CAATCGTCTGTATTTCCATA	N/A
Software and algorithms		
Fiji	Schindelin et al.^67^	https://fiji.sc/
Cell Ranger v7.1.0	Zheng et al.^68^	https://support.10xgenomics.com/single-cell-gene-expression/software/downloads/latest
Scanpy	Wolf et al.^69^	https://github.com/theislab/scanpy
PyDESeq2	Muzellec et al.^70^	https://github.com/owkin/PyDESeq2
STAR v.2.7.9a	Dobin et al.^71^	https://github.com/alexdobin/STAR
Fastp	Chen et al.^72^	https://github.com/OpenGene/fastp
FeatureCounts (Subread v2.0.2)	Liao et al.^73^	http://subread.sourceforge.netge.net/
Bismark	Krueger and Andrews^74^	https://github.com/FelixKrueger/Bismark
methylKit (v1.30.0)	Akalin et al.^75^	https://github.com/al2na/methylKit

### Experimental Model And Study Participant Details

#### Chimpanzee samples

Chimpanzee primary cells were obtained by Kyoto University under approval of the Animal Welfare and Animal Care Committee for Center for the Evolutionary Origins of Human Behavior, Kyoto University (Approval ID: 2016-009; 2018-034) and by the MRC Laboratory of Molecular Biology from Twycross Zoo–East Midlands Zoological Society under approval of the Animal Welfare and Ethical Review Body (AWERB). Blood samples were taken during routine veterinary health check procedures from three animals (Leo, 34 years old male, Kyoto; Pendensa, 41 years old female, Kyoto; TZ-15, 36 years old male, Twycross Zoo). Primary fibroblasts were established from post-mortem skin autopsies from two individuals (Umetaro, 9-years-old, male, Nasu World Monkey Park; Pico, 2-years-old, female, Kyoto).

#### Mouse strains

NSG mice (Jackson Laboratory) were maintained in a biofacility with daily health checks by trained staff. The mice were maintained in a lighting regime of 12:12 hours light:dark with food and water supplied ad libitum. Use of animals in this project was approved by the animal committee for Tokyo Medical and Dental University (Approval ID: A2022-141C2).

#### Cell Culture

Cell lines are listed in the key resources table. Cultures were maintained in humidified incubators at 37°C in 5-7% CO_2_ and for PSCs 5% O_2_. Cells were cultured without antibiotics and confirmed negative for mycoplasma by periodic PCR screening.

### Method Details

#### Cell culture

##### Chimpanzee fibroblasts

Chimpanzee fibroblast cells were cultured in either MEM-alpha (ThermoFisher Scientific, 11900016) or advanced DMEM supplemented with 10% fetal bovine serum (Corning, 35-010-CV). Cells were passaged by dissociation with 0.25% trypsin (TheroFisher Scientific, 25200056). Cultures were used at low passage numbers (<7) for reprogramming.

##### Conventional PSCs

Conventional primed PSCs were propagated in AFX medium^46^ (N2B27 basal medium supplemented with 20 ng/ml Activin-A, 20 ng/ml bFGF, and 2 or 5 μM XAV939) on geltrex-coated dishes or on MEF feeders. ROCK inhibitor (Y-27632, 10μM) was added to media during replating. Cells were passaged by dissociation with 0.25% trypsin or TrypLE every 3-4 days.

##### Naive PSCs

Chemically reset (cR) and directly reprogrammed chimpanzee naïve PSCs were propagated in N2B27 containing PXGL^43^ (PD032590,1 μM; XAV939, 2 μM; Gö6983, 2 μM; human LIF, 10 ng/mL) and further supplemented with 20 ng/mL Activin-A, 20 ng/mL IL6, and 0.5-1 μM EPZ-6438 (PXGL-A6E) or EPZ only (PXGL-E). Cells were routinely expanded on inactivated MEF feeders seeded on 0.1% gelatine-coated plates. Y-27632 (10 μM) and geltrex (Thermo Fisher Scientific, A1413302) were added during replating.^14^ Cells were passaged by dissociation with TrypLE (Thermo, 423201) every 3-4 days. For feeder-free culture, naive PSCs were seeded on geltrex-coated dishes with addition of geltrex and Y-27632 to medium.

##### Reprogramming

Sendai virus vector mediated generation of conventional induced PSCs^76^ was carried out by reprogramming chimpanzee erythroid progenitors (TZ-15) using the Cytotune 2.0 kit (Thermo Fisher, A16517) or PBMCs (Pendensa, Leo) and fibroblasts (Pico, Umetaro) using erasable SeVdp(KOSM302L).^77^ Following vector infection cells were plated in medium for human PSCs. After 10-14 days, iPSC-like colonies were picked manually and replated. After expansion for 2-3 passages, PCR for the Sendai NP protein gene was used to test for absence of erasable SeV. Cultures with undetectable NP protein gene and PSC morphology were expanded. In the case of TZ-15, Ja-C12 was chosen based on competence to form brain organoids.

Alternatively, chimpanzee fibroblast reprogramming was performed using the StemRNA 3rd Gen Reprogramming Kit (ReproCell) as described.^14^ In brief, low-passage dermal fibroblasts were dissociated with trypsin and plated in 4-well dish (ThermoFisher, 144444) at a density of 10,000 cells/cm^2^. The following day, medium was changed to AFX and cells were transfected with NM-RNA reprogramming cocktail using the Lipofectamine® RNAiMAX™ transfection reagent (ThermoFisher, 13778030). Culture medium was refreshed 12 hours after transfection. The transfection process was repeated over 6 days. Cells were maintained in AFX medium to obtain conventional iPSCs or transferred to PXGL-based medium to produce naïve iPSCs.

##### Generation of naïve PSCs by resetting conventional PSCs

Conventional chimpanzee PSCs were dissociated with 0.25% trypsin or TrypLE and seeded on MEF-coated plates at 1-3 x 10^4^ cells/cm^2^ in AFX medium supplemented with 10 μM Y-27632 (day-1). The next day (day 0), medium was exchanged to cRM-1, N2B27 supplemented with 1μM PD0325901 (PD), 10 ng/ml human LIF (in-house), and 1 mM valproic acid sodium salt (VPA, Sigma, P4543).^41^ From day 2 to 3, when extensive cell death became apparent, the medium was changed to PXGL-A6E. Around day 10-14, refractile rounded colonies were observed. Cells were dissociated with 0.25% trypsin or TrypLE, then replated on MEF-coated plates at 1:2 to 1:5 split ratio with 10 μM Y-27632. Colonies were live-stained with conjugated SUSD2 antibody (Bio-legend) to monitor naïve-like identity.^43^

##### Genetic modification

To establish PRC2 knock-out cells, CRISPR guide sequences from published reports^38,60^ (Table S1) were inserted in the CML32.2 vector (U6-gRNA-PGK-puro). CML32-gRNA plasmids were mixed with TrueCut™ Cas9 Protein v2 (Thermoscientific, A36499) and transfection performed using the Neon™ system.

A GFP-reporter was introduced into chimpanzee naïve PSCs by co-transfection of pBase and LTR-GFP-zeo plasmids. Two days after transfection, zeocin (50 μg/ml) was applied and selection maintained for one week. The population was expanded thereafter in PXGL-E and remained GFP positive.

##### Human naïve PSC culture

Human naive PSCs, embryo-derived HNES1^12^ and directly reprogrammed naïve iPSCs,^14^ were propagated in PXGL on MEF as described.^43^ For feeder-free culture, cells were cultured in PXGL supplemented with EPZ or GSK126 as indicated. Medium was topped up daily and cultures passaged every 4 days with addition of geltrex and Y-27632 on replating.

#### Differentiation

##### Capacitation

Transition to somatic lineage competence was performed following the capacitation process described for human naive PSCs.^46^ Naïve PSCs were cultured on geltrex coated plates without feeders for one passage then replated at a 1:6 split ratio. The following day, medium was changed to N2B27 supplemented with 2 μM XAV with daily renewal thereafter. After 10 days capacitation, cells were dissociated with 0.5 mM EDTA and passaged into AFX medium for expansion.

##### Somatic lineage induction

For neuroectoderm induction by dual SMAD inhibition,^78^ naïve or capacitated cells were plated in geltrex pre-coated wells at 5,000 cells/cm^2^. Culture medium was changed to N2B27 supplemented with 1 μM A83-01 and 500 nM LDN193189 (LDN) at day1. Cells were cultured for 10 days with daily medium changes.

For paraxial mesoderm induction,^79^ cells were passaged in geltrex pre-coated wells at 5,000 cells/cm2. The next day, culture medium was changed to N2B27 basal medium supplemented with 3 μM CHIR99021 and 500 nm LDN for 2 days. From day 3, 20 ng/ml FGF2 was added to the medium and continued culture for another 4 days.

For definitive endoderm induction,^80^ cells were dissociated and plated in geltrex pre-coated wells at 10,000 cells/cm^2^. One day after plating, the culture medium was changed to CDM2 supplemented with 100 ng/ml Activin A, 100 nM PI-103, 3 μM CHIR99021, 10 ng/ml FGF2, 3 ng/ml BMP4 and 10 μg/ml heparin. From day 2, BMP4 was replaced by 250 nM LDN and culture continued for two days.

##### Teratoma formation

PSCs were suspended in ice-cold Matrigel and injected into testes of 8 weeks old male NSG mice at ~ 5 x 10^5^ cells per site. Animals were sacrificed after 60 days and teratomas were collected. Teratomas were fixed in 4%PFA and embedded in paraffin. Sections were stained with Haematoxylin and Eosin for histological inspection.

##### Trophectoderm induction and trophoblast differentiation

Naïve PSCs were passaged once geltrex-coated plates to remove feeders. They were then replated at a split ratio of 1:6 in PXGL without EPZ. The next day, culture medium was changed to N2B27 plus 3 μM PD03 and 3 μM A83-01 (PD+A83). Cells were treated for 5 days with daily medium changes.

For cytotrophoblast (CT) expansion, day3 TE cells were transferred to ACE medium (N2B27 supplemented with 1 μM A83, 2 μM CHIR and 50 ng/ml EGF).^55^ The culture medium was refreshed every two days. To enrich for CT cells, cultures at passage 2 were dissociated with accutase for 5 minutes and rinsed in wash buffer (DMEM-F12 with 0.1% BSA), resuspended gently in ACE medium and filtered through a 40 μm filter. Cell clusters retained on the filter were washed gently with culture medium. After three washes, cell clusters were harvested and exapnded on geltrex-coated dishes in ACE medium as described.^55^

For extravillous trophoblast (EVT) differentiation,^55^ CT cells were dissociated with accutase and seeded on geltrex-coated plates at 5,000 cells/cm^2^. The following day, ACE medium was replaced by EVT-1 medium, comprising DMEM-F12 with 0.1 mM 2ME, 0.3% BSA, 1% IST-X supplement, 4% KSR, 7.5 μM A83, 2.5 μM Y-27632 and 100 ng/ml NRG1. Geltrex was added at plating. On day 3, the culture medium was changed to EVT-2 (EVT-1 medium without NRG1). At day 6, cells were dissociated into single cells and passaged 1:2 into a fresh geltrex-coated well. The next day, culture medium was changed to EVT-3 (EVT-2 medium without KSR). At day 8, EVT cells were collected for RNA extraction or fixed for immunostaining.

For syncytiotrophoblast (ST) differentiation,^55^ CT cells were plated as above then transferred to ST medium (DMEM-F12 supplemented with 4% KSR, 2 μM forskolin, 0.1mM 2ME, 0.3% BSA, 2.5 μM Y-27632, 1% ITS-X supplement). Medium was refreshed at day 3 and cells cultured for another 3 days before staining.

##### Blastoid formation

For blastoid formation,^20,21^ naïve cells were first cultured with PXGL-A6E in non-adherent dishes for three days. Floating colonies were collected and transferred to PALY medium (N2B27 with 2 μM PD, 1 μM A83, 1 μM LPA and 10 μM Y-27632). After 24 hours, cell clusters were transferred to ALY (N2B27 with 1 μM A83, 1 μM LPA and 10 μM Y-27632). After a further 24 hours, immature blastoids with small cysts become apparent. Blastoids were then transferred to N2B27 and culture continued for a further 24-30 hours. Alternatively, dissociated naïve PSCs were dispensed directly in ultra-low attachment multi-well plates (Corning Costar) in PALY at 50-200 cells/well and centrifuged to form clusters. After 36 h, aggregates were transferred individually into non-adherent U-bottomed 96-well plates (Greiner) containing pre-warmed ALY. The following day, medium was changed to N2B27 for a further 24 hours.

#### Immunostaining

For live cell staining, conjugated antibodies were diluted 1:100 in culture medium and applied to cells for 1 hour before observation under a fluorescence microscope. For fixed cell staining, cells were washed with PBS and fixed with 4% PFA for 30 min, then incubated with blocking solution (1% BSA, 2% donkey serum in PBS) for 2 hours. For nuclear antigens, 0.1% Triton was added for permeabilization. Cells were incubated with primary antibody (1:300 dilution) for 2 hours at room temperature (or 4°C overnight) followed by three washes and 1 h incubation with secondary antibody (1:1000 dilution). DAPI (1:3000 dilution) was applied to visualise nuclei.

Blastoids were fixed with 4% PFA in PBS for 15 min at room temperature. Samples were rinsed in PBS containing 3 mg/mL polyvinylpyrrolidone (PBS/PVP) and permeabilized with PBS/PVP containing 0.25% Triton X-100 for 30 min. Blocking was performed in embryo blocking buffer comprising PBS supplemented with 0.1% BSA, 0.01% Tween20 and 2% donkey serum for 2-3 hour at 4°C. Samples were incubated in blocking buffer with 500 ng/mL DAPI for 1 hour at room temperature in the dark. DAPI-stained samples were rinsed three times for 15 min in blocking buffer. Antibodies are listed in key resources table.

#### Chromosome analysis

Metaphase spreads were prepared after colcemid treatment and imaged using a DMI800 microscope for chromosome counting. G-banded karyotype analysis was performed by contract karyotyping (Nihon Gene Research Laboratories, Japan, or Cell Guidance Systems, UK).

#### Immunoblotting

Cells cultured in 6-well plates were scraped and collected in PBS. Cells were washed with PBS then resuspended with lysate buffer (RIPA buffer supplemented with proteinase inhibitor, phosphatase inhibitor and Benzonase) and incubated on ice for 30 minutes. Cell lysates were centrifuged at 4°C, 12,000 g/min for 30 minutes. Supernantants were collected and stored at -20°C. Thawed samples were denatured in loading buffer (Thermoscientific, NP0007) with reducing agent then fractionated by SDS-PAGE. Following semi-dry transfer, membranes were washed with 1xTBST and blocked in 5% BSA for 2 hours at room temperature. After blocking, the membrane was incubated with primary antibody (1:1000 dilution) overnight at 4°C. Membrane was then washed with TBST three times and incubated with secondary antibody (1:3000 dilution) for 2 hours at room temperature. After incubation, the membrane was washed with TBST for three or four times then developed with chemiluminescent substrate (Thermoscientific, 34577). Antibodies are listed in key resources table.

#### Reverse transcription PCR

RNA samples were extracted using ReliaPrep™ RNA Cell Miniprep System (Promega, Z6010). cDNA was synthesized using GoTaq® Probe qPCR and RT-qPCR Systems (Promega, A6101). Data are from biological duplicates or triplicates. PCR primers are listed in the key resources table.

#### Flow cytometry

Cell sorting and flow analysis were performed using SH800 (SONY), FACSARIA III (BD), and CytoFLEX (Beckman Coulter) instruments. To purify reset naïve-like PSCs, cultures propagated for 2-3 passages in PXGL-AE6 were dissociated into single cells using 0.25% trypsin or TrypLE, stained with APC or PE conjugated-SUSD2 antibody (Biolegend), and the SUSD2 high expressing fraction collected and replated.

#### Transcriptome sequencing

##### Bulk RNA sequencing

Total RNA was extracted from cultures using TRIzol/chloroform, followed by RNA precipitation with isopropanol. Genomic DNA was depleted using TurboDNAse and clean-up was performed using Zymogen Clean and Concentrator kit. RNA integrity assessed by Tapestation using RNA Screen Tape, and concentration measured using Qubit RNA High Sensitivity reagent. Ribosomal RNA was depleted from 1 μg of total RNA using Ribozero. Sequencing libraries were prepared using the NEB Next Ultra Library prep kit for Illumina. Sequencing was performed on the Novaseq S2 platform. Reads were trimmed using Fastp^72^ to remove sequencing adapters and low quality (<Q22) bases from the 3’ end. Reads shorter than 75bp were discarded. Trimmed reads were then aligned to the ENSEMBL Mouse and Chimpanzee references (ENSEMBL_Mus_musculus.GRCm39.109 and ENSEMBL_Pan_troglodytes.-Pan_tro_3.0-rel109) using STAR^71^ version=2.7.9a. The aligned reads were processed by XenofilteR^81^ version 1.6 to remove transcripts of suspected mouse origin.^61^

##### 10X Genomic single cell RNA sequencing of PSCs

Samples were dissociated and labelled using the 3’ CellPlex kit Set A (10X Genomics 1000261), then multiplexed and single cells isolated using the Chromium Next Gem Chip G Single Cell kit (10X Genomics 1000120) on the Chromium X. Libraries were prepared using the Chromium Next GEM Single Cell 3’ v3.1 kit (10X Genomics 1000269) with 3’ Feature Barcode Kit (10X Genomics 1000262). Library quality and quantity was assessed using High Sensitivity D5000 ScreenTape on a 4200 Tapestation system (Agilent). Sequencing was performed on the Illumina NovaSeq 6000 using the S2 Reagent Kit v1.5.

##### Blastoid SmartSeq2 scRNA-seq

Individual day 4 blastoids were collected in droplets of N2B27 and rinsed twice with N2B27. Mural trophectoderm was excised with glass needles under the dissecting microscope. ICMs (with polar trophectoderm) and mural trophectoderm were moved to separate droplets of trypLE. ICMs were incubated at 37°C for 10 min, mural trophectoderm for 20 min. After dissociation, single cells were flash-frozen on dry ice in SMART-Seq HT sorting solution (Takara Bio 634439) and stored at -80°C for up to 2 weeks. Illumina-compatible sequencing libraries were prepared using the Takara SMART-Seq mRNA Single Cell LP kit (Takara Bio 634788) and Unique Dual Index Kits (Takara Bio 634752, 634753, 634754, 634755) according to the manufacturer’s protocol. cDNA and library quality were assessed using the High Sensitivity D5000 ScreenTape (Agilent 5067-5592 and 5067-5593) on a 4200 Tapestation (Agilent G2991BA). Sequencing was performed with paired end 150bp reads on the Illumina NovaSeq 6000 using the S1 Reagent Kit v1.5 300 cycles (Illumina 20028317). Reads were trimmed using FastP^72^ then aligned to the P.trog genome Pan_tro_3.0 with annotation file 109 using STAR^81^ v2.7.10. Count matrices were generated using the GenCount feature within STAR v2.7.10.

#### Whole genome bisulfite sequencing (WGBS)

Post-bisulfite adaptor tagging (PBAT) libraries for whole-genome DNA methylation sequencing were prepared from purified genomic DNA according to the “High throughput” protocol^82^ with the following modifications. 100 ng of isolated gDNA was used for bisulfite conversion using the EZ DNA Methylation-Gold Kit (Zymo, D5005). First strand synthesis was performed with a biotinylated First-Strand Primer and the final incubation was extended to 90 min. For the second-strand synthesis, a modified Second-Strand Primer (CAGACGTGTGCTCTTCCGATCTNNNNNNNNN) was used, allowing for standard TruSeq dual indexed sequencing. The final incubation was extended to 90 minutes. The final PCR amplification (8 cycles) was performed with standard Illumina TruSeq dual-indexed primers. Libraries were sequenced on the NovaSeq 6000 platform.Raw sequence reads were processed using the nf-core/methyl-seq v2.6.0 pipeline (doi: 10.5281/zenodo.1343417), part of the nf-core collection of reproducible bioinformatics workflows.^83^ The analysis was conducted within containerized environments provided by Bioconda^84^ and Biocontainers,^85^ ensuring computational reproducibility. The pipeline was executed using Nextflow v24.04.2.^86^ Alignment and quantification were performed with Bismark,^74^ employing the ‘pbat’ option for library type and ‘local’ alignment mode. Paired-end reads were aligned to the Pan_tro_3.0 reference genome, focusing exclusively on autosomal chromosomes.

### Quantification and Statistical Analysis

#### qRT-PCR and cell number counts

Data are presented as mean ± SD from biological replicates. The number of replicates is stated in the associated figure legends.

#### Quantification of 5-methylcytosine by LC-MS/MS

The Monarch Genomic DNA Purification kit (New England Biolabs #T3010) was used to isolate DNA from cell pellets with elution in LC-MS grade water. DNA was subsequently enzymatically digested into nucleosides as described.^87^ The nucleosides were injected into an Agilent 1290 Infinity UHPLC instrument with a ZORBAX Eclipse Plus C18 Rapid Resolution HD column (2.1x100 mm, 1.8 μm, Agilent #959758-902), connected to an Agilent 6495B triple quadrupole mass spectrometer operating in positive mode. The chromatographic method and mass spectrometer parameters are described elsewhere.^87^ Data were quantified in MassHunter Quantitative Analysis for QQQ (v 10.1) using standard curves and heavy labelled internal standards for each analysed nucleoside.^54,87^ The lower limit of quantification was 0.25 fmol for 5mdC and 1 fmol for dC and dG. The limit of detection was 0.025 fmol for 5mdC, 0.5 fmol for dC and 0.1 fmol dG.

#### Transcriptome analyses

##### Bulk RNA-seq

Naïve and primed chimpanzee PSC samples from this study and published human PSC samples^18,51^ were combined into a single matrix using the intersection of named protein coding gene orthologs. Differential gene expression analysis between naïve and primed culture conditions was performed using PyDESeq2^70^ with standard workflow and parameters. For heatmap visualisations gene expression values were log_2_ normalised and z-score transformed.

##### TP53 mutation analysis

Sequencing adapters were trimmed and low quality (<Q22) bases removed using Fastp^72^ before alignment to mouse (ENSEMBL_ Mus_musculus.GRCm39.109) or chimpanzee (ENSEMBL_Pan_troglodytes.Pan_tro_3.0.109) reference genomes using STAR^71^ version=2.7.11b with –outSAMattributes NH HI AS nM NM option for XenofilteR. Aligned reads were filtered by XenofilteR^81^ version=1.6. Reads mapped surrounding mouse Trp53 (chr11:69,468,307-69,485,577) or chimpanzee TP53 (chr17:7,938,025-7,965,596) regions were extracted, sorted and indexed using SAMtools^88^ and loaded on IGV^89^ version=2.18.4.

Using IGV, we confirmed that chimpanzee reads were mapped well to chimpanzee TP53, and filtered chimpanzee TP53 reads were not contaminated by mouse Trp53-derived reads. The reference genome sequence contains unidentified N sequences even in coding regions and some exon annotations are missing. We therefore manually searched for variants in samples from three naïve and primed PSC lines at different passages.

##### naive PSC scRNA-seq

Demultiplexing and alignments were performed using Cell Ranger 7.1.0.^68^ Scanpy was used to read and analyse raw read counts from the Cell Ranger output. Cells expressing fewer than 5000 genes, more than 9000 genes, or more than 20% mitochondrial reads were filtered out. Contaminating MEF cells, identified by expression of Vimentin (VIM), were removed before further analysis. The resultant count matrix was normalised and log-transformed. Initial dimensionality reduction was carried out using PCA and taking the top 25 PCs prior to non-linear dimensionality reduction using UMAP (neighbours = 30, min_dist = 0.1). For UMAP expression plots, gene expression values were log_2_ normalised. For heatmap visualisations, gene expression values were log_2_ normalised followed by maximum normalisation by dividing the expression values of each gene by their maximum observed expression.

##### Blastoid scRNA-seq

Samples were processed using the standard Scanpy single cell analysis pipeline. Data were normalised and log transformed using the default Scanpy commands. Low quality cells were filtered by removing samples with total counts <4000 or number of genes per counts <8000, leaving 185/217 (85%) samples. Genes that were present in less than 20 samples were removed from downstream analysis, leaving 15085/29314 genes. A UMAP calculated using the top 50 principal components of the expression data was generated with nearest neighbors and min_dist parameters of 30 and 0.1 respectively. Unsupervised clustering of the UMAP embedding was obtained via Leiden clustering with a resolution parameter of 1 (default).

##### Projection of PSC samples on human embryo reference

To relate bulk or single cell PSC samples to human embryo development we took the UMAP model object^48^ and used the umap.-transform function on the log_2_ transformed counts of each dataset to position samples into the UMAP latent space.

##### Correlation distance metrics analysis

Pseudo-bulk samples were generated for each labelled group in the human embryo UMAP^48^ by calculating the mean expression of each gene. Samples were subset to the 3012 genes selected to generate the embryo UMAP and normalised via log_2_ transformation followed by scaling each gene to values between 0-1. Pairwise correlation distance metrics were calculated for each human pseudo-bulk and chimpanzee sample and scaled between 0-1 to aid interoperability.

##### DNA methylation analyses

CpG methylation calls were filtered and normalized using the methylKit R package (v1.30.0),^75^ with the top 0.1% of read counts removed to control for outliers. For clustering and methylation distribution analysis in the combined sample group, only CpGs with a minimum coverage of 3× were used. Replicates were pooled for the combined analysis, while CpGs with a minimum coverage of 5× were retained for the replicate-specific analysis.

Promoter regions were defined as spanning from -900 to +100 base pairs (bp) relative to the transcription start site (TSS) of protein-coding genes, based on Ensembl release 112 annotations. Methylation analysis of these promoters included all CpG sites with at least 1× sequencing coverage. After pooling replicate samples, we applied the regionCounts function from the methylKit package, setting a minimum coverage threshold of 2×. For downstream analysis, only promoters with more than 5 methylation counts were selected. Gene ontology (GO) analysis was conducted using the R package gprofiler2,^90^ with the human gene ontology database. The significance threshold was set at a p-value < 0.01 and a q-value < 0.05, using Bonferroni correction.

Human imprinted control regions (ICRs) taken from a previous study^41^ were mapped to the chimpanzee genome using UCSC’s Liftover tool^91^ with the hg38ToPanTro5.over.chain file. Liftover regions that were separated by less than 250 bp were merged into a single ICR. To standardize these regions for methylation quantification, they were resized to 2000 bp, centered, and defined as 1000 bp on either side of the midpoint. Only CpGs with at least 3× coverage were included, and replicates were pooled before applying the regionCounts function from methylKit, with a minimum of 2× coverage. ICRs with more than 5 methylation counts were selected for further analysis.

## Supplementary Material

**Supplemental Information**

Supplemental information can be found online at https://doi.org/10.1016/j.stem.2025.02.002.

Document S2

Figures S1-S4

Table S1

## Data Availability

Raw sequencing data are deposited in Sequence Read Archive (SRA: PRJNA1086168) and processed bulk RNA-seq, scRNA-seq and WGBS datasets in Gene Expression Omnibus (GEO: GSE282157, GSE278810 and GSE264735).

## Highlights

- Inhibition of PRC2 to reduce H3K27me3 enables expansion of chimpanzee naive PSCs
- Chimpanzee naive PSCs closely match human naive PSCs and early embryo epiblast
- Chimpanzee naive PSCs form blastoids with appropriate cell-type identities
- PRC2 inhibition facilitates feeder-free propagation of human naive PSCs
