# Supplementary material for: Inhibition of PRC2 enables self-renewal of blastoid-competent naive pluripotent stem cells from chimpanzee
Source: Cell Stem Cell. Author manuscript; Available in PMC 2025 Jul 4. (PMC7617839; doi:10.1016/j.stem.2025.02.002)
Supplement: Figures S1-S4 [file EMS206528-supplement-Figures_S1_S4.pdf]

Cell Stem Cell, Volume *32*

Supplemental Information

Inhibition of PRC2 enables self-renewal of blastoid-competent naive pluripotent stem cells from chimpanzee

Tao Huang (黄瑫), Arthur Radley, Ayaka Yanagida, Zhili Ren (任志丽), Francesca Carlisle, Somayyeh Tahajjodi, Dongwan Kim, Paul O'Neill, James Clarke, Madeline A. Lancaster, Zoe Heckhausen, Jingran Zhuo (卓靖然), João Pedro Agostinho de Sousa, Petra Hajkova, Ferdinand von Meyenn, Hiroo Imai, Hiromitsu Nakauchi, Ge Guo (郭歌), Austin Smith, and Hideki Masaki

SUPPLEMENTAL INFORMATION

Table S2 Details of chimpanzee naïve PSC lines

Information on naïve PSC lines generated and stably expanded for at least 10 passages.

Table S2 Details of chimpanzee naïve pluripotent stem cell lines

Animal	Age; Sex	Somatic Cells	Primary reprogramming	Naive line	Naïve PSC Generation	Modal chromosomes	G-banded karyotype	Feeder-free	PXGL-E#
Pico	2yr; female	Dermal fibroblasts	RNA	CP-R1	Resetting	48 (P11)			
			RNA	CP-127	Resetting	48 (P23)		>10 passages	8 passages no feeders
			N/A	TCP1	Direct reprogramming	48 (P10)			5 passages
			SeVdp	Pico#16-cR	Resetting	48 (P10)	P10; 48,XX[20/20]		
Umetaro	9yr; male	Dermal fibroblasts	RNA	CPU6-R1	Resetting	48 (P20)	P20; 48,XY[20/20]	>10 passages	
			SeVdp	Ume#6-cR	Resetting	48 (P8)	P8; 48,XY[20/20]		
Pendensa	41yr; female	PBMC‡	SeVdp	Pen#23-cR	Resetting	48 (P35)	P35; 48,XX[20/20]		
Leo	34yr; male	PBMC‡	SeVdp	Leo#9-cR	Resetting	48 (P8)	P8; 48,XY[20/20]		
TZ-15	36yr; male	Erythroid progenitors	SeV (CytoTune)	JB-R1	Resetting	48 (P8)	P11; 48,XY,del(3)(p?)[7]/48,XY[13]		

# Culture in PXGL with EPZ alone.

‡ Peripheral blood mononuclear cells

Figure S1 Generation and characterization of chimpanzee naive PSCs. Related to Figure 1 and Figure 2

A. Immunostaining of general pluripotency factors in chimpanzee primed iPSCs in AFX medium. Bar=278.5 μm.
B. Immunostaining of naive pluripotency markers in day 7 reset iPSCs in PXGL. Bar=278.5 μm.
C. G-banded karyotypes for female and male reset PSCs at P10 and P8 respectively.
D. Metaphase spread from feeder-free naive PSCs at P10.
E. Immunostaining of naïve PSCs at P8 without feeders. Bar=278.5 μm
F. qRT-PCR analysis of feeder-free naive PSCs. SD from biological triplicates.
G. qRT-PCR analysis of lineage markers after differentiation of capacitated PSCs to definitive endoderm (DE), neuroectoderm (NE), and paraxial mesoderm (PM). SD from biological triplicates.
H. Histological sections of teratomas formed by chimpanzee PSCs. Bar=100 μm.
I. PCA of bulk RNA-seq data for chimpanzee naive and primed PSCs.
J. Projection on the human embryo UMAP embedding of published bulk RNA-seq datasets for human naive and primed PSCs.
K. Immunostaining of H3K27me3 and H2AK119ub in primed male and female cells and in naïve female cells cultured with and without EPZ. Bar=278.5 μm.
L. Relative 5mdC level of chimpanzee primed, naive and capacitated PS cells detected by LC/MS-MS.
M. Methylation percentages of human Imprinting Control Regions (ICRs) mapped to the chimpanzee genome using Liftover, centered and extended by 2kb.

Figure S1

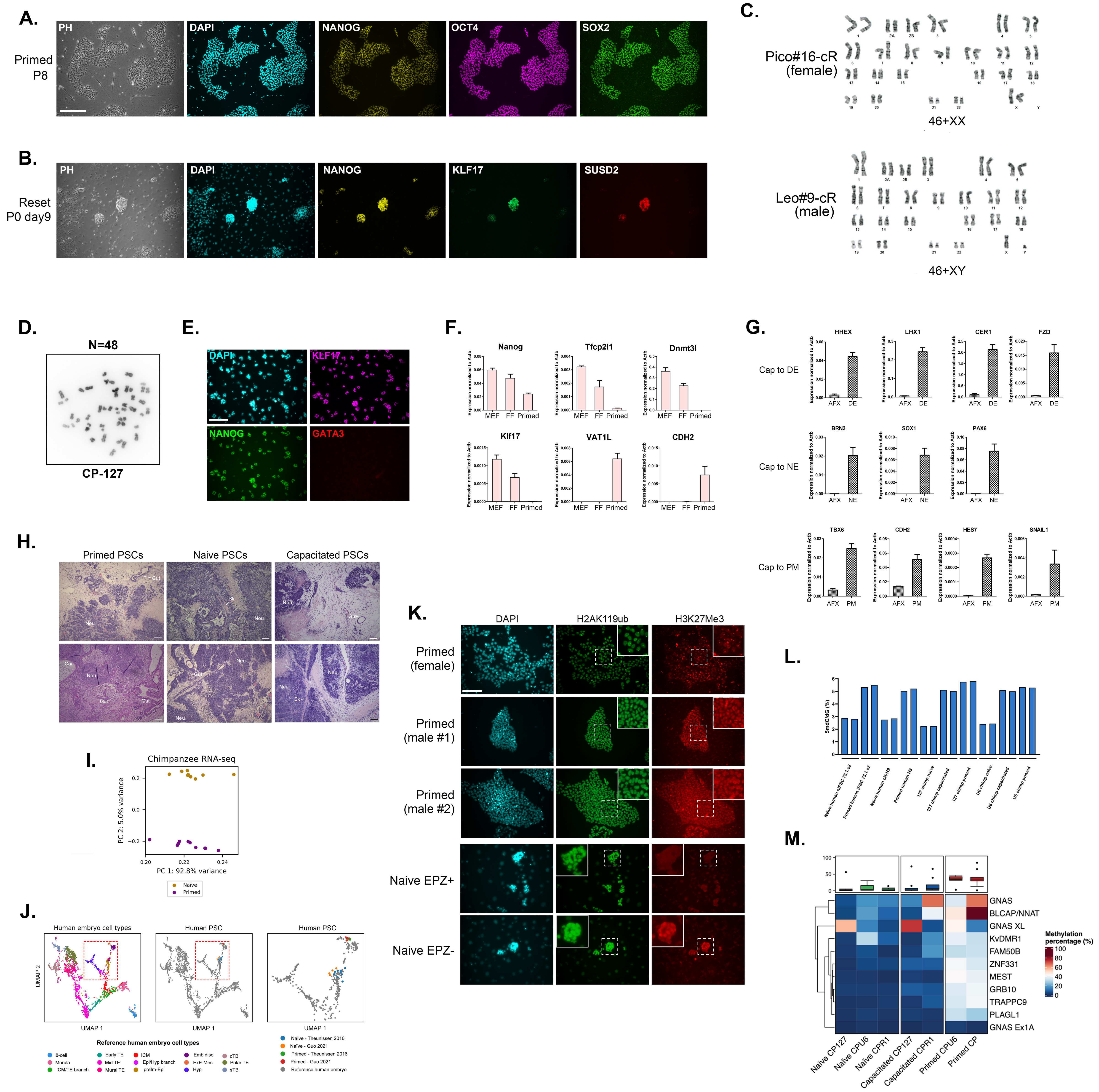
A.
Primed P8
PH
DAPI
NANOG
OCT4
SOX2
B.
Reset P0 day9
PH
DAPI
NANOG
KLF17
SUSD2
C.
Pico#16-cR (female)
46+XX
Leo#9-cR (male)
46+XY
D.
N=48
CP-127
E.
DAPI
KLF17
NANOG
GATA3
F.
Nanog
Tfcp2l1
Dnmt3l
Klf17
VAT1L
CDH2
MEF FF Primed
G.
Cap to DE
HHEX
LHX1
CER1
FZD
AFX DE
Cap to NE
BRN2
SOX1
PAX6
AFX NE
Cap to PM
TBX6
CDH2
HES7
SNAIL1
AFX PM
H.
Primed PSCs
Naive PSCs
Capacitated PSCs
I.
Chimpanzee RNA-seq
PC 2: 5.0% variance
PC 1: 92.8% variance
Naive
Primed
J.
Human embryo cell types
Human PSC
UMAP 1
UMAP 2
Reference human embryo cell types
Naive - Theunissen 2016
Naive - Guo 2021
Primed - Theunissen 2016
Primed - Guo 2021
Reference human embryo
K.
DAPI
H2AK119ub
H3K27Me3
Primed (female)
Primed (male #1)
Primed (male #2)
Naive EPZ+
Naive EPZ-
L.
5mdCdG (%)
M.
GNAS
BLCAP/NNAT
GNAS XL
KvDMR1
FAM50B
ZNF331
MEST
GRB10
TRAPPC9
PLAGL1
GNAS Ex1A
Methylation percentage (%)
Naive CP127
Naive CPU6
Naive CPR1
Capacitated CP127
Capacitated CPR1
Primed CPU6
Primed CP

Figure S2 Extraembryonic differentiation and blastoid formation by chimpanzee naive PSCs. Related to Figure 3

A. Immunostaining on day 5 of trophectoderm differentiation of CP-127 cells after 8 passages without feeders. Bar=278.5 µm.
B. Schematic of cytotrophoblast (CTB) generation and immunostaining.
C. Immunostained images of syncytiotrophoblast and extravillous trophoblast differentiation. Bar=278.5 µm.
D. qRT-PCR analysis of syncytiotrophoblast (ST) and extravillous trophoblast (EVT) differentiation from cytrophoblast (CT)
E. Immunostaining of hypoblast cells differentiated from naive PSCs. Bar=278.5 µm.
F. Phase and immunostaining images of day 4 blastoids formed from Pen#23-cR cells in microwells. Bar=100 µm.
G. Frequencies of cavity formation and presence of GATA4 positive cells together with GATA3 and OCT4 positive cells for Pen#23-cR blastoids. SD from biological triplicates.
H. UMAP clustering of scRNA-seq data from blastoids showing selected makers of epiblast, hypoblast, trophectoderm and amnion.
I. qRT-PCR analysis of naive marker expression in reset or directly reprogrammed naïve iPSCs.
J. Immunostaining on day 5 of trophectoderm induction from directly reprogrammed TCP1 naive iPSCs at passage 10. Bar=278.5 µm.
K. qRT-PCR analysis of markers of trophoblast and amnion (GABRP) upon differentiation of directly reprogrammed naive iPSCs. SD of biological duplicates.
L. UMAP clustering of scRNA-seq data from directly reprogrammed naive PSCs on feeders or feeder free with expression of selected naïve pluripotency regulators.
M. Clustermap of scRNA-seq samples from directly reprogammed or reset naive PSCs with or without feeders (indicated in colour scheme) for a set of known naive, primed and trophectoderm markers. Normalised expression.
N. Separate projections onto the human embryo UMAP embedding of scRNA-seq data from reset or directly reprogrammed chimpanzee naive PSCs cultured on feeders or feeder-free. Sample colours as in M.

Figure S2

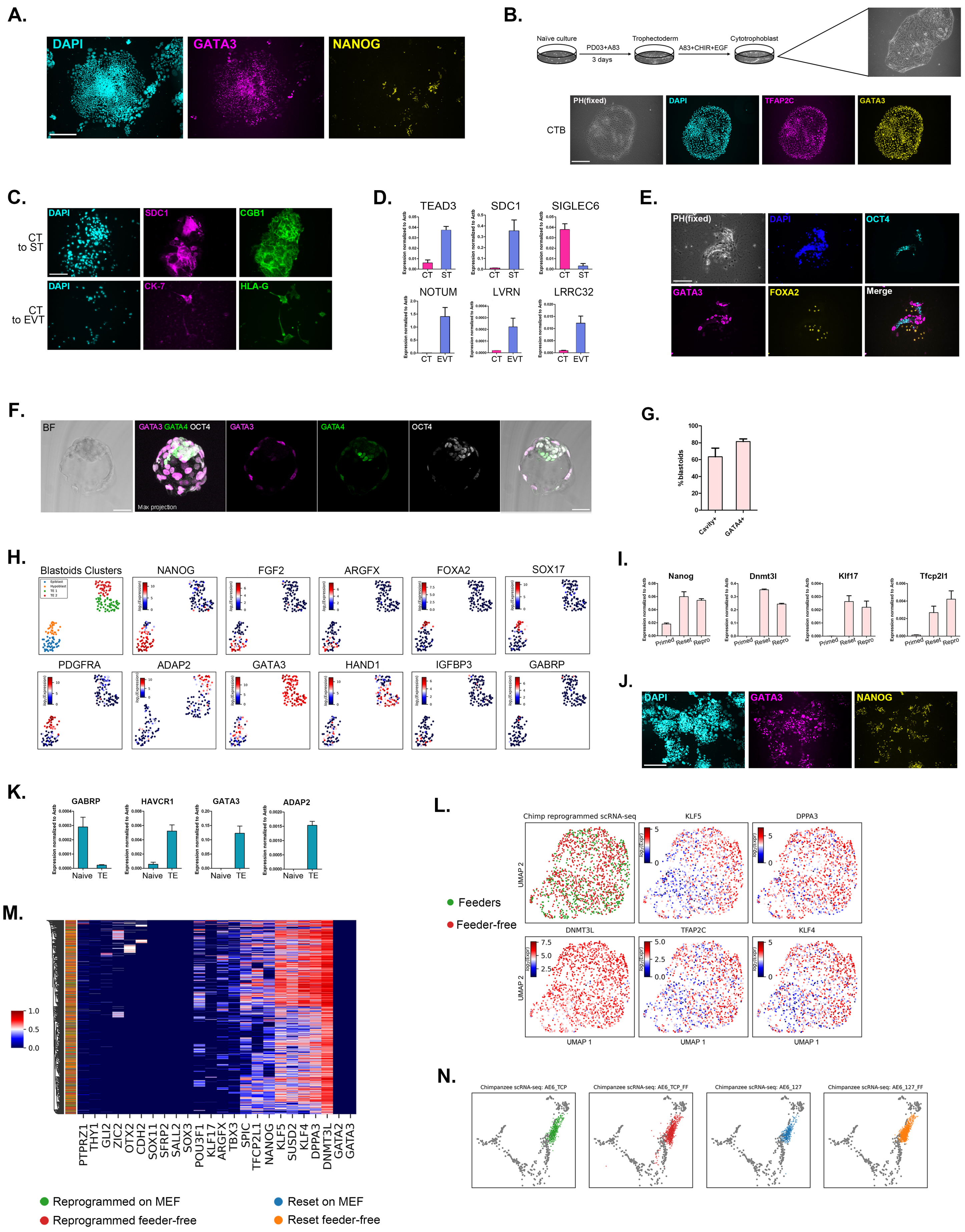

Figure S3 Dependence of chimpanzee naive PSC self-renewal on inhibition of PRC2. Related to Figure 4

A. Morphology and SUSD2 staining of chimpanzee naive PSCs cultured in PXGL-E. Bar=144 µm.
B. qRT-PCR analysis of naïve PSCs in different conditions and induced trophectoderm. SD of biological duplicates.
C. Morphology of newly reset PSCs at P2 in PXGL-A6E or 4CL. Bar=278.5 µm
D. SUSD2 flow analysis of reset cells as in C.
E. Quantitation of SUSD2 flow analysis in indicated concentrations of EPZ.
F. SUSD2 flow analysis of established naive PSCs cultured with in PXGL-A6 with different EZH2 inhibitors.
G. Immunostaining of EZH2, EED and SUZ12 in respective KO cell lines maintained in PXGL without EPZ for 1 or 5 passages. Bar=278.5 µm.
H. Immunostaining of naive markers in expanded PRC2-KO cell lines. Bar=278.5
I. qRT-PCR analysis of markers in trophoblast differentiated from wildtype and PRC2-KO naïve PSCs. SD of biological duplicates.
J. Phase and fluorescence images of EZH2-KO cells mixed 50:50 with GFP-labelled cells and cultured for 5 passages in PXGL-E. Bar=287.5µm.

Figure S3

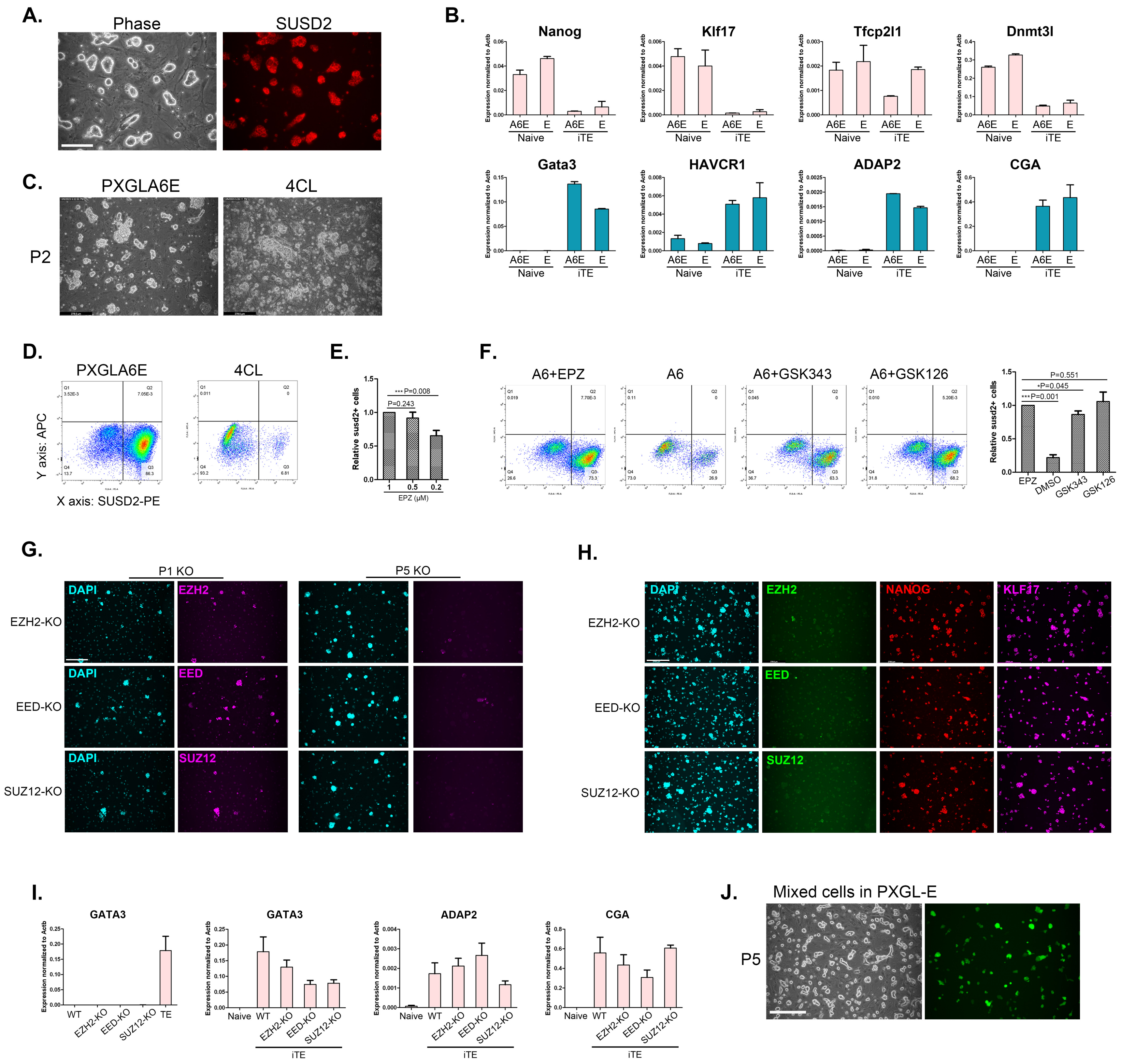

A.
Phase
SUSD2
B.
Nanog
Klf17
Tfcp2l1
Dnmt3l
Gata3
HAVCR1
ADAP2
CGA
Expression normalized to Actb
A6E E A6E E
Naive iTE
C.
PXGLA6E
4CL
P2
D.
PXGLA6E
4CL
Y axis: APC
X axis: SUSD2-PE
E.
Relative susd2+ cells
P=0.008
P=0.243
EPZ (μM)
F.
A6+EPZ
A6
A6+GSK343
A6+GSK126
P=0.551
P=0.045
P=0.001
EPZ DMSO GSK343 GSK126
G.
P1 KO
P5 KO
EZH2-KO
EED-KO
SUZ12-KO
DAPI
EZH2
EED
SUZ12
H.
DAPI
EZH2
EED
SUZ12
NANOG
KLF17
I.
GATA3
ADAP2
CGA
WT EZH2-KO EED-KO SUZ12-KO TE
Naive WT EZH2-KO EED-KO SUZ12-KO
iTE
J.
Mixed cells in PXGL-E
P5

Figure S4 Feeder-free culture of human naive PSCs in medium with EZH2 inhibition. Related to Figure 4

A. Feeder-free culture of human naive PSCs in PXGL with or without GSK126 (n=3)
B. Phase contrast and immunostained images of human naive PSCs cultured on MEF without EPZ or feeder-free with EPZ.
C. SUSD2 flow analysis of cultures as in B. Primed PSC sample is negative control.
D. qRT-PCR analysis of naive marker expression in feeder-free human PSCs maintained with or without EPZ. SD from technical duplicates.
E. GATA3-mKO2 reporter expression following induction of trophoblast with PD+A83 for 5 days from naive PSCs expanded without feeders in PXGL-E for 8 passages.
F. qRT-PCR analysis of trophoblast markers after PD+A83 treatment of feeder-free naive PSCs as in E. SD from technical duplicates.
G. Projection on the human embryo embedding of sc-RNA-seq data from indicated human naïve PSCs cultured on feeders (MEF) or feeder-free with EPZ (FF).
H. Clustermap as in S2L of human scRNA-seq samples cultured on feeders or feeder-free with EPZ for three passages.

Figure S4

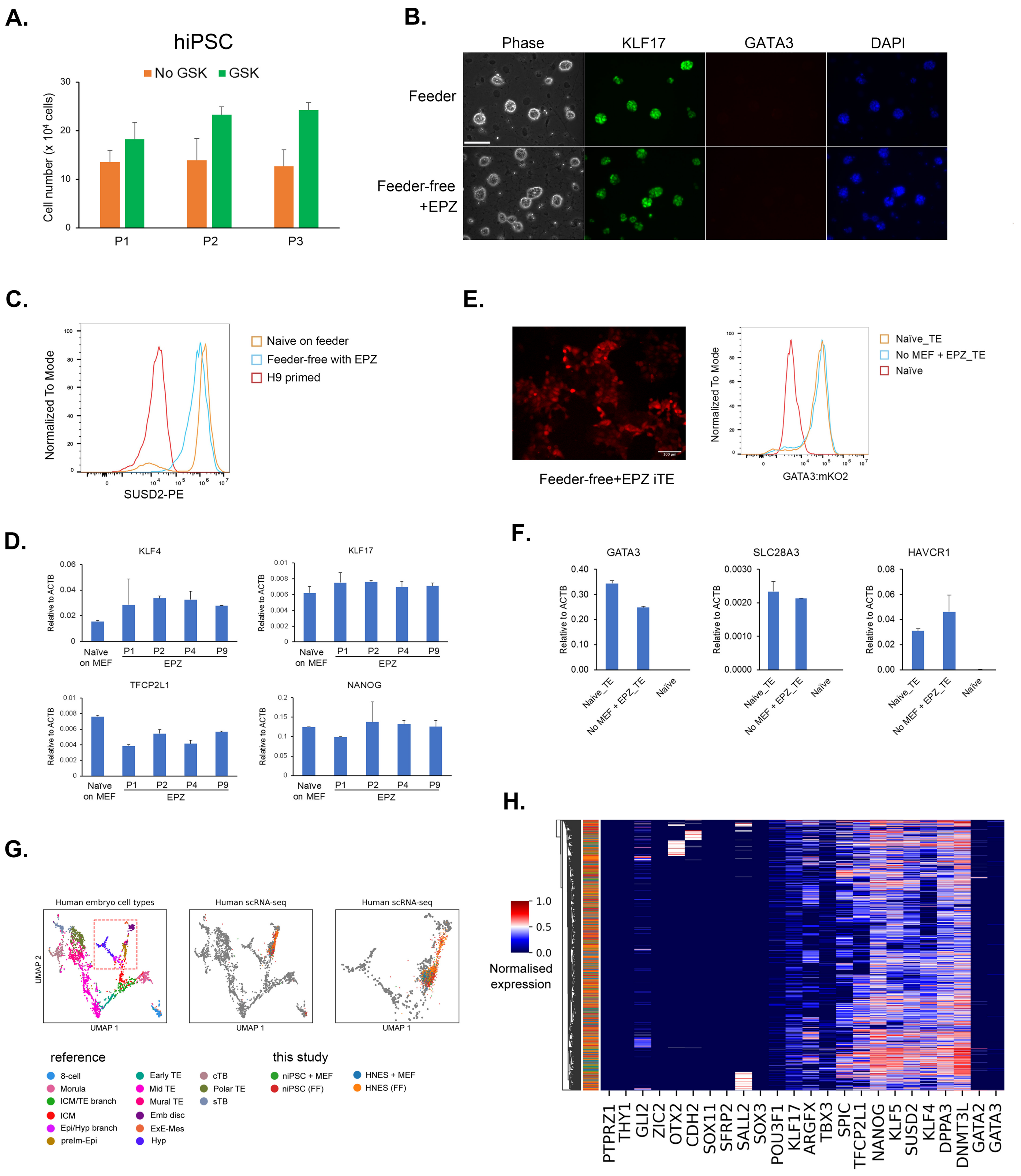
A.
hiPSC
No GSK
GSK
Cell number (x 10⁴ cells)
P1
P2
P3
B.
Phase
KLF17
GATA3
DAPI
Feeder
Feeder-free +EPZ
C.
Normalized To Mode
SUSD2-PE
Naive on feeder
Feeder-free with EPZ
H9 primed
E.
Feeder-free+EPZ iTE
GATA3:mKO2
Naïve_TE
No MEF + EPZ_TE
Naïve
D.
KLF4
KLF17
TFCP2L1
NANOG
Relative to ACTB
Naïve on MEF
P1
P2
P4
P9
EPZ
F.
GATA3
SLC28A3
HAVCR1
G.
Human embryo cell types
Human scRNA-seq
UMAP 1
UMAP 2
reference
8-cell
Morula
ICM/TE branch
ICM
Epi/Hyp branch
preIm-Epi
Early TE
Mid TE
Mural TE
Emb disc
ExE-Mes
Hyp
cTB
Polar TE
sTB
this study
niPSC + MEF
niPSC (FF)
HNES + MEF
HNES (FF)
H.
Normalised expression
PTPRZ1
THY1
GLI2
ZIC2
OTX2
CDH2
SOX11
SFRP2
SALL2
SOX3
POU3F1
KLF17
ARGFX
TBX3
SPIC
TFCP2L1
NANOG
KLF5
SUSD2
KLF4
DPPA3
DNMT3L
GATA2
GATA3
